# Supplementary material for: Morc3 silences endogenous retroviruses by enabling Daxx-mediated histone H3.3 incorporation
Source: Nat Commun. 2021 Oct 14;12:5996. doi: 10.1038/s41467-021-26288-7 (PMC8516933; doi:10.1038/s41467-021-26288-7)
Supplement: Supplementary file 1 — Supplementary Information [file 41467_2021_26288_MOESM1_ESM.pdf]

Morc3 silences endogenous retroviruses by enabling Daxx-mediated histone H3.3 incorporation (Groh et al.)

Supplementary information

Supplementary Figures

**Figure S1.**	Comparison of the SHIN screen with Chelmicki et al. and FACS gating strategy.
**Figure S2.**	Morc3-3xFLAG knock-in strategy and validation of knock-in.
**Figure S3.**	Validation of knock-out and rescue cell lines.
**Figure S4.**	Morc3 rescues gene and ERV expression.
**Figure S5.**	Characterization of Morc3 dependent chromatin changes.
**Figure S6.**	Morc3 dependent chromatin changes on selected Morc3 targets.
**Figure S7.**	Chromatin changes in Morc3 knock-out cells can be rescued.
**Figure S8.**	Validation of Morc3 mutant rescue cell lines.
**Figure S9.**	Morc3 can associate with H3K4me3 promoters.
**Figure S10.**	Morc3 mutants fail to rescue Morc3-dependent chromatin changes.
**Figure S11.**	Morc3 mutant proteins fail to rescue increased chromatin accessibility on Morc3 targets.
**Figure S12.**	Nuclear Daxx levels are not changed in Morc3 mutant rescue cell lines.
**Figure S13.**	Histone H3.3 ChIPseq analysis.
**Figure S14.**	Morc3 mutant proteins fail to rescue histone H3.3 enrichment on Morc3 target genes.
**Figure S15.**	Regulation of Morc3 target ERVs and genes in Daxx and H3.3 ko cells.
**Figure S16.**	Morc3 target genes are similarly regulated in Daxx, H3.3 and Morc3 ko cells.

Supplementary Tables

**Table S1.**	Plasmids
**Table S2.**	sgRNA oligonucleotides
**Table S3.**	Cell lines
**Table S4.**	Antibodies
**Table S5.**	RT-qPCR oligonucleotides
**Table S6.**	Oligonucleotides for cloning
**Table S7.**	Oligonucleotides for library preparation of sgRNA screen
**Table S8.**	Primers used for ATAC-seq from Buenrostro, et al (2013)
**Table S9.**	Sequencing files
**Table S10.**	Software

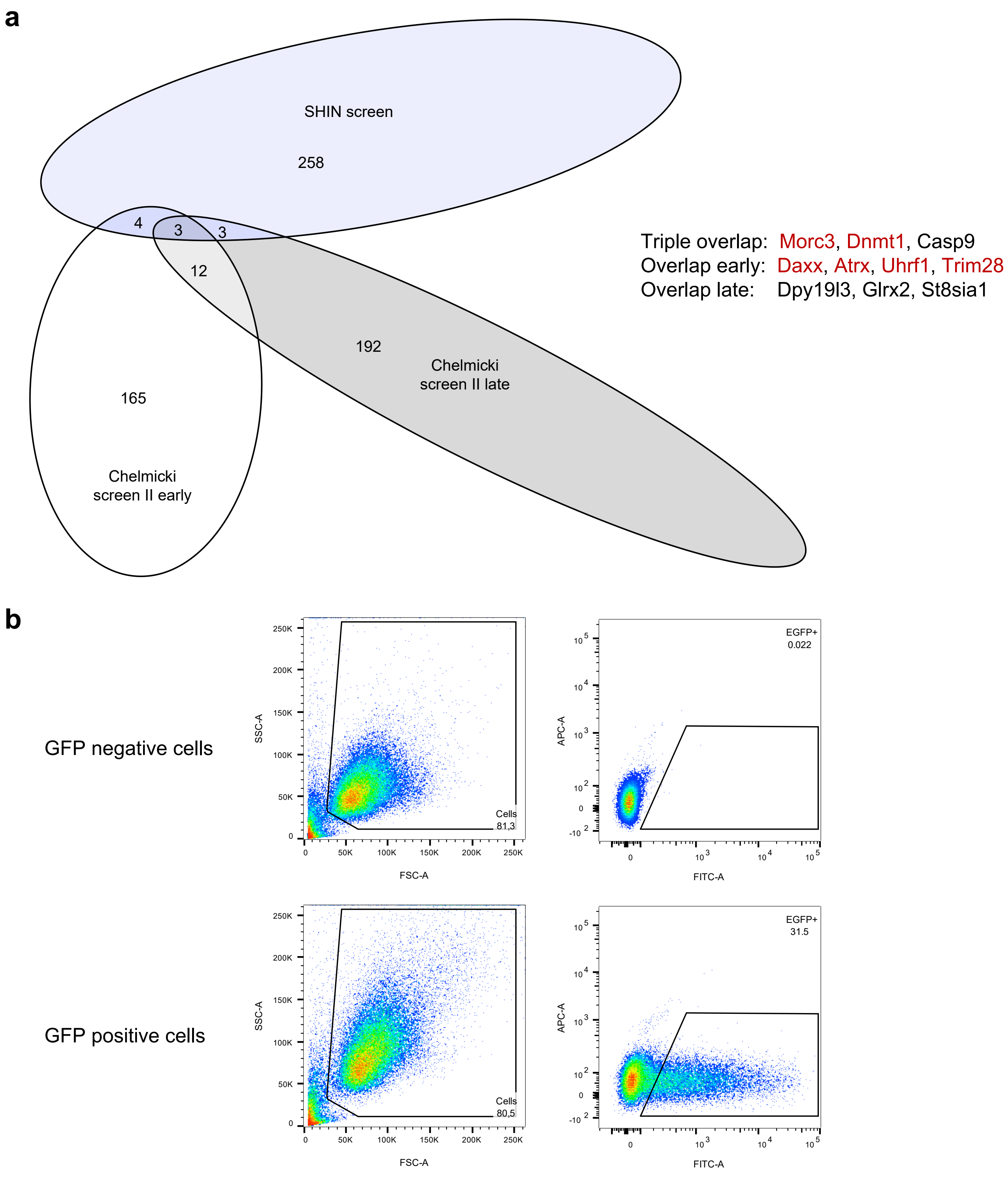

Figure S1. Comparison of the SHIN screen with Chelmicki et al. and FACS gating strategy. a Comparison of screening hits between our Morc3 SHIN screen and an IAP based screen from Chelmicki et al.[1] Venn Diagram of top hits (provided p- values < 0.01) from early and late Chelmicki et al. screen II and Morc3 SHIN screen is shown. Overlapping genes between all three screens, overlap between Morc3 and early screens, and overlap between Morc3 and late screens are specified. Known ERV silencing factors are marked in red. **b** The FACS gating strategy for SHIN reporter experiments consists of gating for live cells by forward (FSC) and side scatter (SSC) followed by gating for negative and positive according to control cells without GFP expression in the FITC-A (GFP) and APC-A (empty) channels.

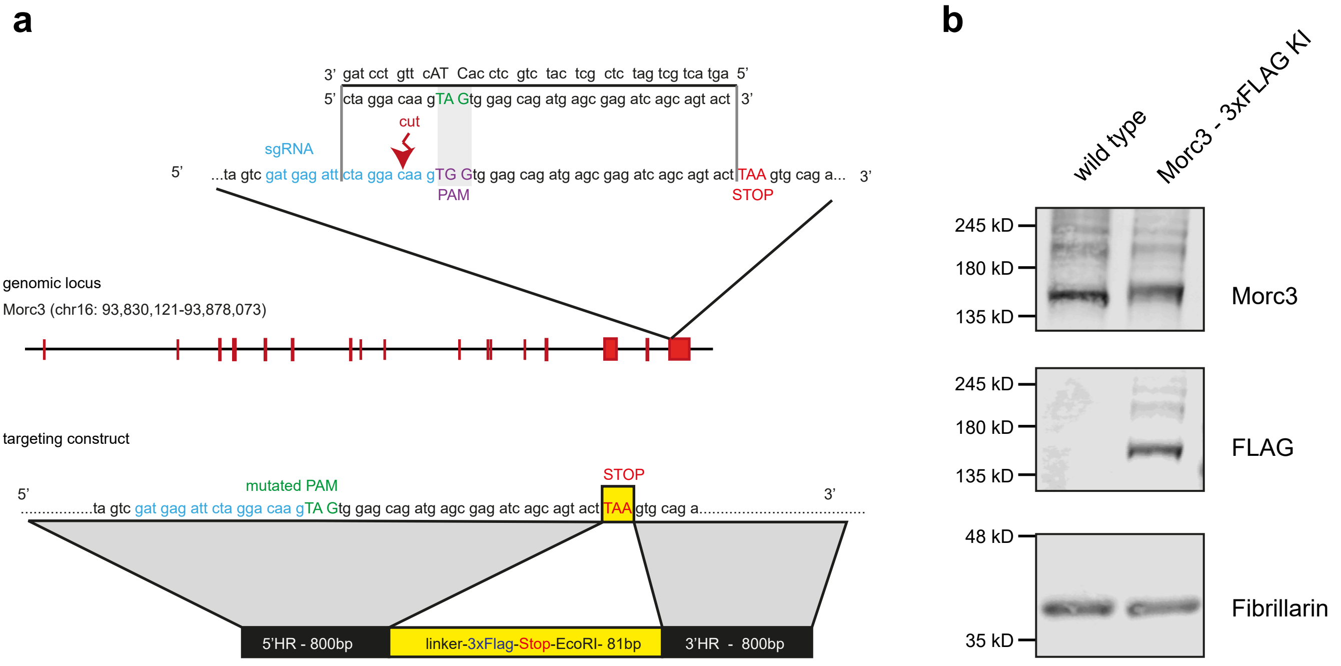

Figure S2. Morc3-3xFLAG knock-in strategy and validation of knock-in. a Schema of the Morc3 genomic locus and the sequence used for sgRNA-mediated double strand break induction to allow for homology-dependent repair with the targeting construct. **b** Western blot analysis of wild type and Morc3 3xFLAG knock-in nuclear extracts using antibodies against Morc3, FLAG and Fibrillarin (loading control). Detection on the same blot with the LI-COR system. Uncropped blot in Source Data.

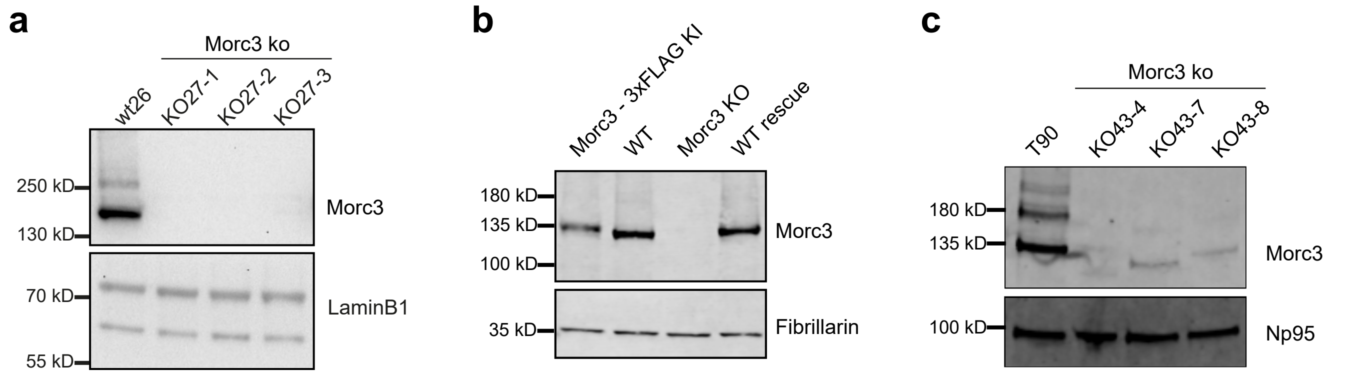

Figure S3. Validation of knock-out and rescue cell lines. a Western blot analysis of wild type vs. Morc3 knock-out clones. Morc3 antibody staining indicates loss of Morc3 expression in the knock-out clones. LaminB1 serves as loading control. **b** Western blot analysis of Morc3 3xFLAG knock-in, wild type, Morc3 ko, and Morc3 rescue cell lines using antibodies against Morc3 and Fibrillarin (loading control).**c** Western blot analysis of Morc3 knock-out clones, based on T90 ES cells. Morc3 antibody staining indicates loss of Morc3 expression in the knock-out clones. NP95 serves as loading control. Detection on the same blot with the LI-COR system. Uncropped blots in Source Data.

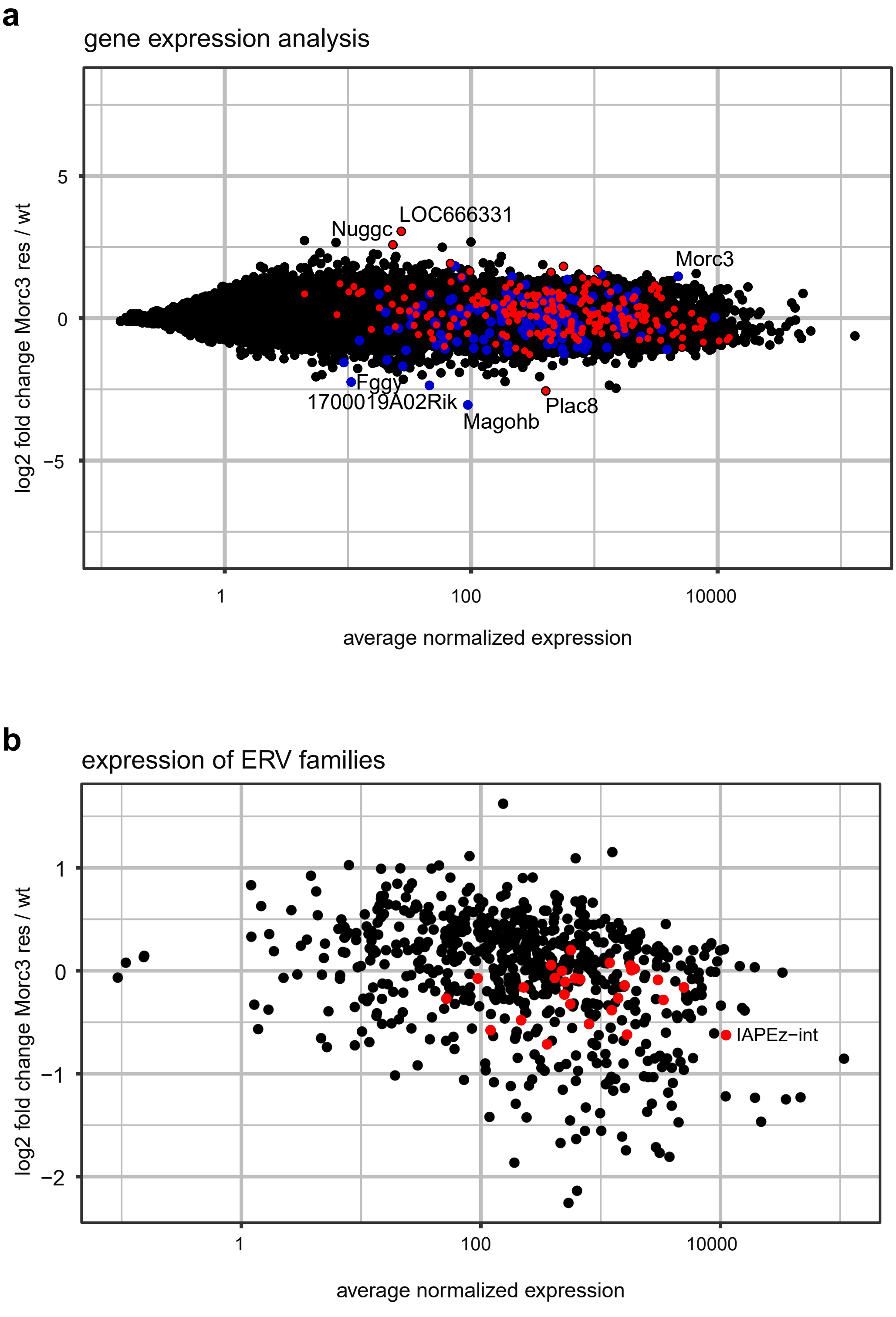

Figure S4. Morc3 rescues gene and ERV expression. **a** Dot plot showing average expression vs. log2-fold change of coding genes in wild type vs. Morc3 rescue ES cells. Colored dots indicate genes with significantly changed expression in the Morc3 ko (**Figure 3a**). Positions of relevant genes are indicated. **b** Dot plot showing average expression vs. log2-fold change of ERV families in wild type vs. Morc3 rescue ES cells. Colored dots indicate ERvs with significantly changed expression in the Morc3 ko (**Figure 3f**). Positions of relevant ERV families are indicated.

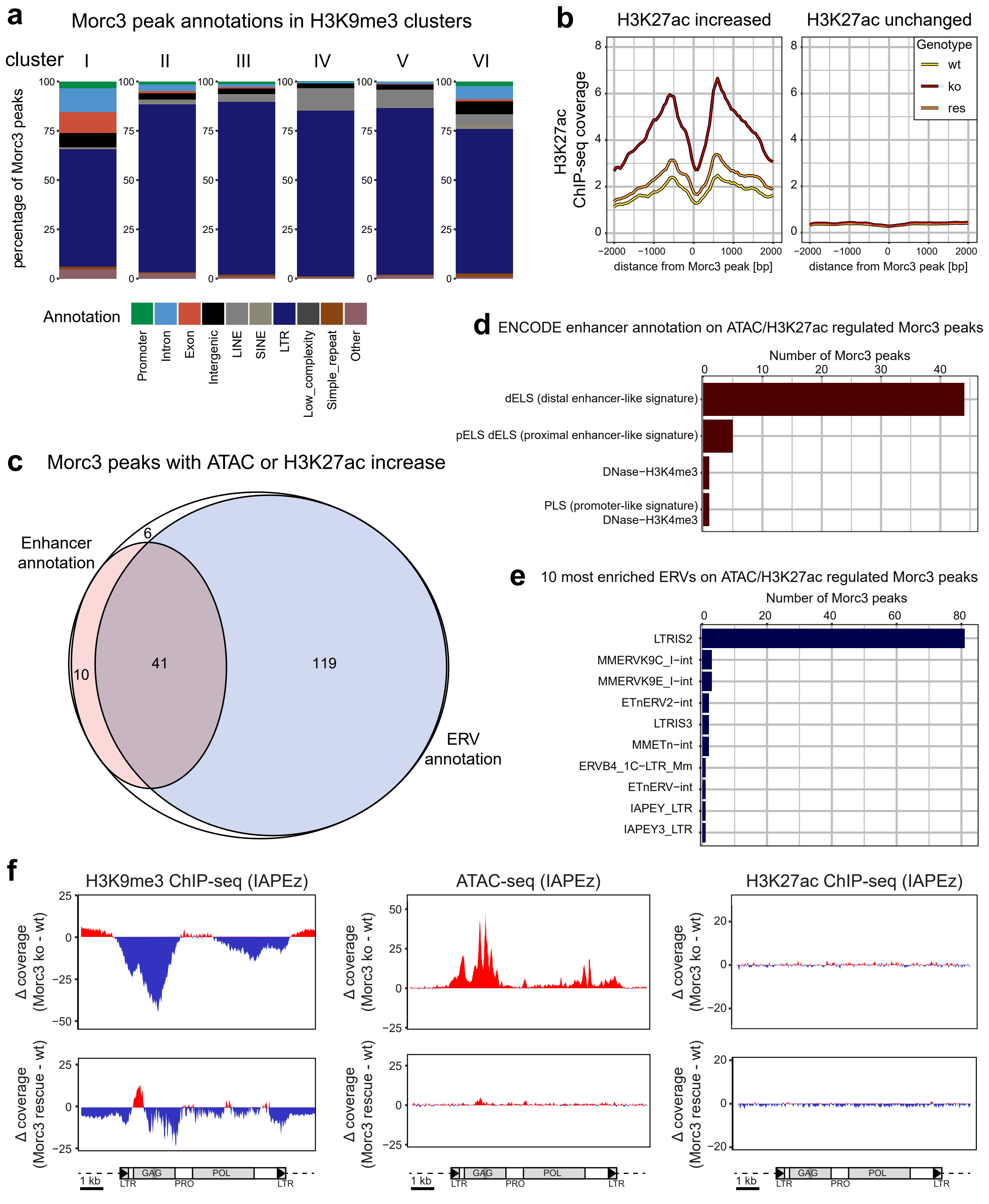

Figure S5. Characterization of Morc3 dependent chromatin changes. a Annotation statistics of Morc3 peak clusters from Figure 4a. **b** Density plot showing the average occupancy of H3K27ac on Morc3 peaks. Left panel shows Morc3 peaks with increased H3K27ac, right panel shows Morc3 peaks with unchanged H3K27ac in Morc3 ko ES cells. **c** Venn diagram showing the overlap between Morc3 peaks with increased accessibility and/or increased H3K27ac in Morc3 ko with ENCODE CRE and ERV annotations. **d** Bar graph depicts number of ATAC/H3K27ac regulated Morc3 peaks with different ENCODE enhancer annotations. **e** Bar graph depicts number of ATAC/H3K27ac regulated Morc3 peaks associated with the 10 most enriched ERV families.

Continued legend Figure S5. f Reduced H3K9me3, increased chromatin accessibility and unchanged H3K27ac on IAPEz elements in Morc3 ko ES cells. Differences in cumulative coverage between Morc3 ko and wild type ES cells (top panel) or Morc3 rescue and wild type ES cells (lower panel) for H3K9me3 ChIP-seq, ATAC-seq and H3K27ac ChIP-seq are plotted. Reduced H3K9me3 and increased accessibility is mainly in the 5'UTR and GAG region. The position of the SHIN sequence is indicated as gray bar.

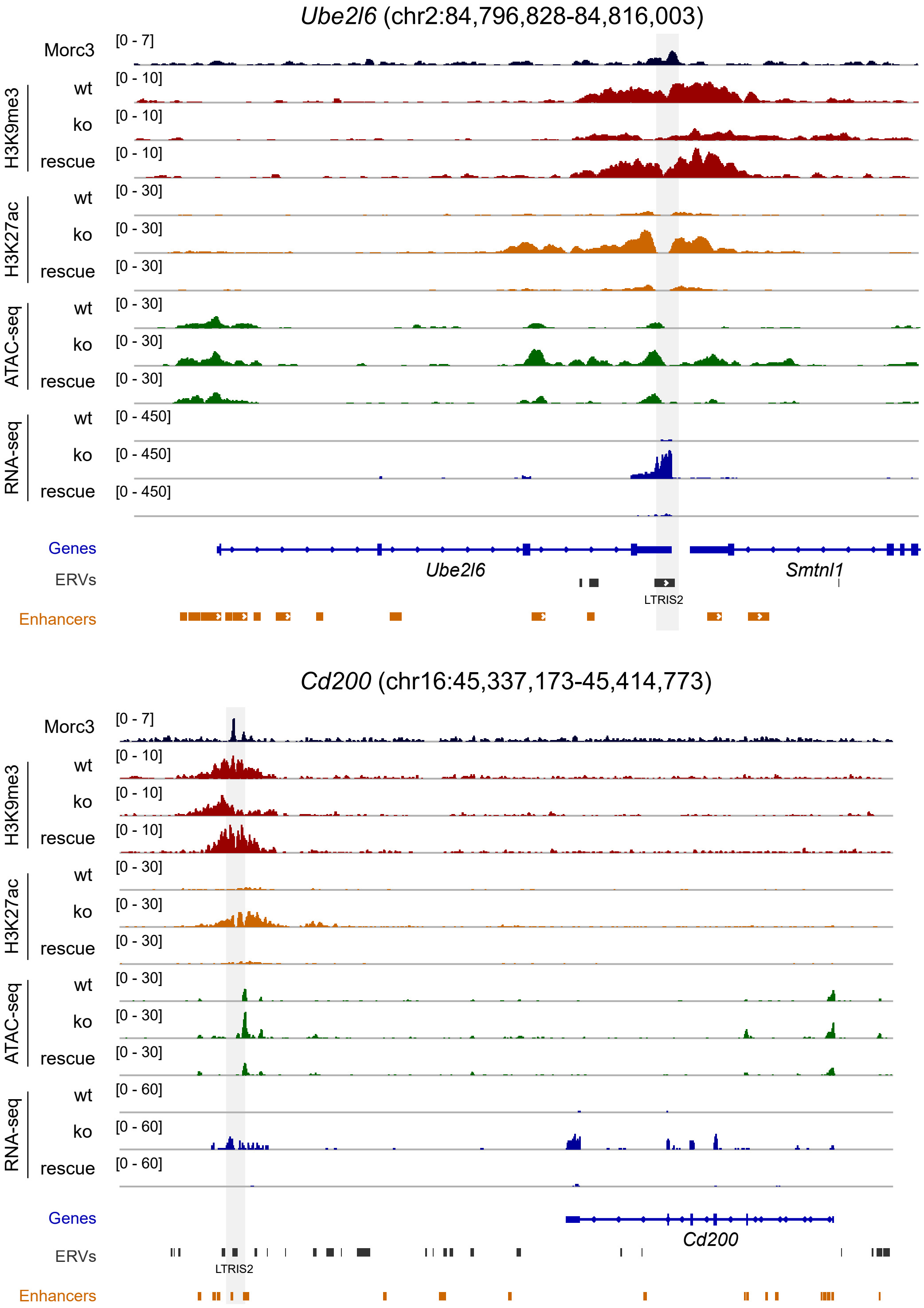

Figure S6. Morc3 dependent chromatin changes on selected Morc3 targets. Genome browser view of Morc3-dependent chromatin changes on target genes (*Ube2l6*, *Cd200*). Positions of Morc3 peaks are indicated by gray boxes. Chromatin and transcriptional changes are rescued in Morc3 rescue ES cells.

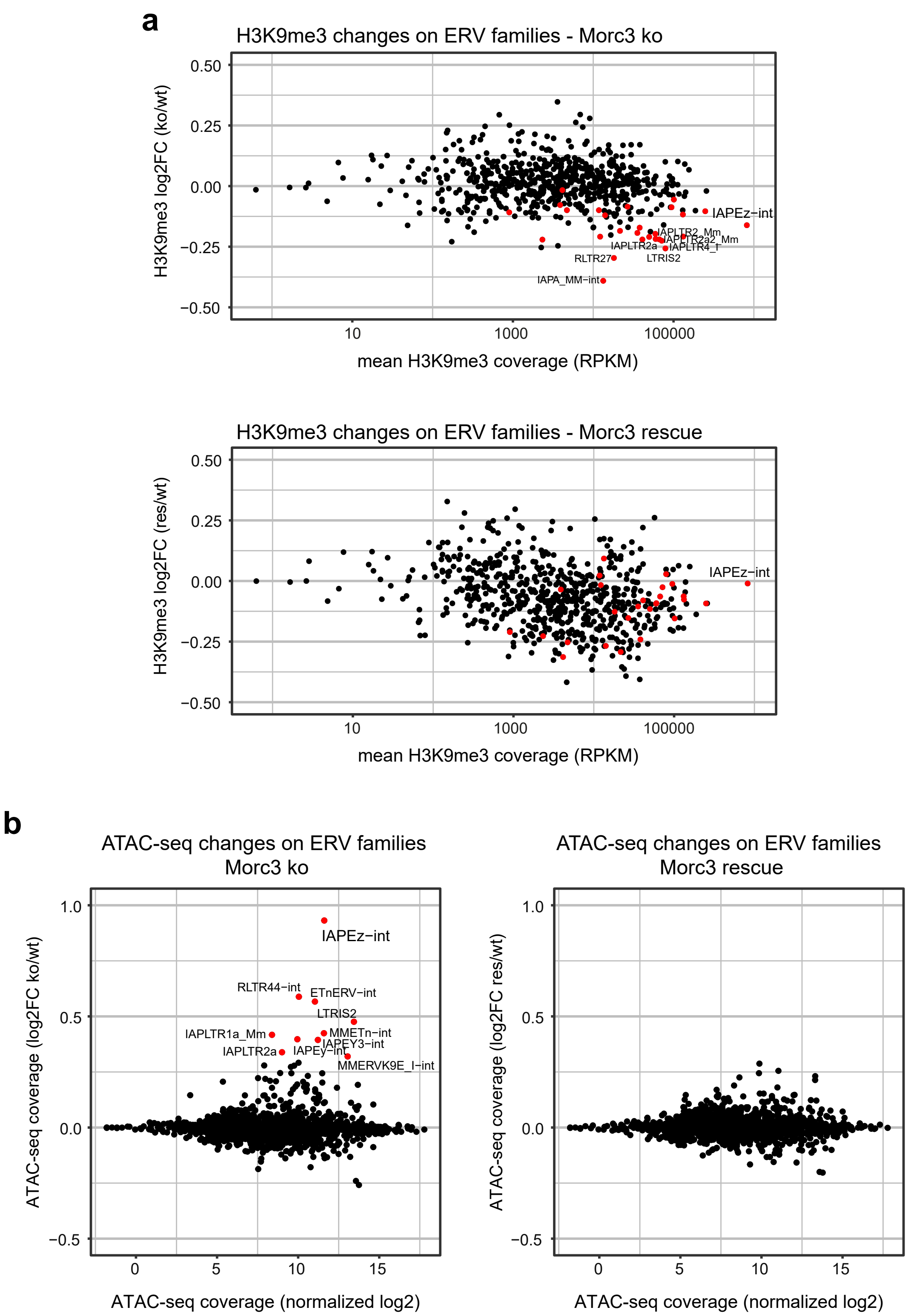

Figure S7. Chromatin changes in Morc3 knock-out cells can be rescued. a Dot plot showing normalized H3K9me3 ChIP-seq coverage vs. log2-fold change of ERV families in wild type vs. Morc3 knock-out ES cells (upper panel) and wild type vs. Morc3 rescue ES cells (lower panel). Red dots indicate ERV families with significantly reduced coverage in Morc3 ko cells (adjusted p-value < 0.05, by DEseq2, n=2 for wt and ko, n=3 for res). H3K9me3 of most families is normalized in Morc3 rescue cells (e.g. IAPEz). **b** Dot plot showing average ATAC-seq coverage vs. log2-fold change of ERV families in wild type vs. Morc3 knock-out ES cells (left panel) and wild type vs Morc3 rescue ES cells (right panel. Colored dots indicate ERV families with significantly increased (red dots) or decreased (blue dots) coverage (adjusted p-value < 0.05, by DEseq2, n=3 for each condition).

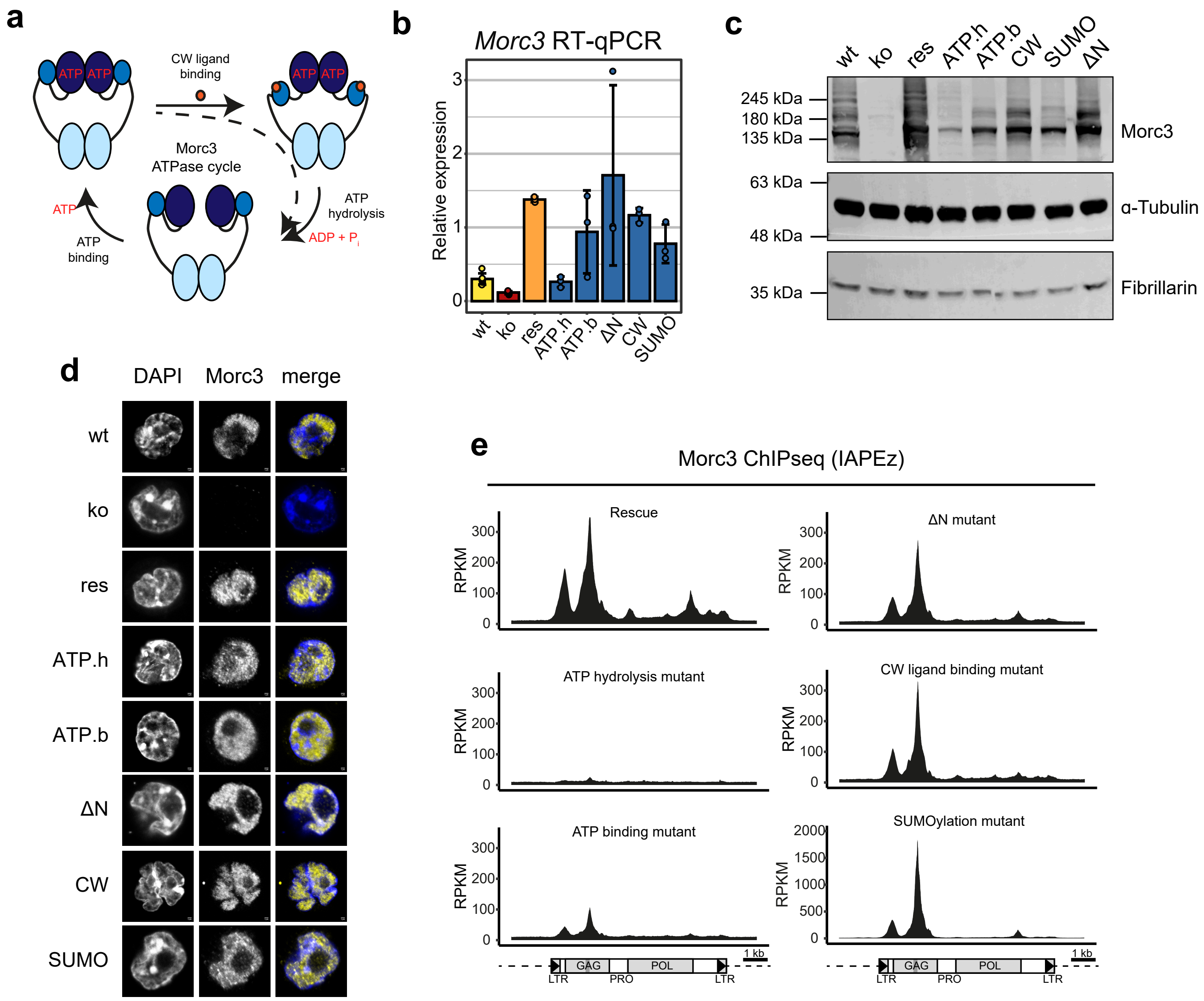

Figure S8. Validation of Morc3 mutant rescue cell lines. a Schema of the Morc3 ATPase cycle. **b** RT-qPCR analysis of *Morc3* expression in Morc3 mutant rescue ES cells. Bar graph depicts mean relative expression to control genes (*Actin* and *Hprt*). Error bars indicate standard deviation of replicate experiments (n=3). **c** Western blot analysis of whole cell lysates from wild type, Morc3 ko, and Morc3 rescue cell lines using antibodies against Morc3 and the loading controls alpha-Tubulin and Fibrillarin. Detection on the same blot with the LI-COR system. Uncropped blots in Source Data. **d** Immunofluorescence analysis of wild type and Morc3 mutant rescue cells. In wild type cells, Morc3 displays a broad nuclear staining, which is lost in Morc3 knock-out cells. All Morc3 mutant proteins display a similar staining pattern as the wild type protein. **e** Cumulative ChIP-seq coverage of Morc3 wild type and mutant proteins on IAPEz elements. Prominent enrichment is over the 5'UTR and the GAG region. The position of the SHIN sequence is indicated as dark gray bar. The ATP hydrolysis mutant does not display significant coverage on IAPEz elements and the ATP binding mutant shows reduced enrichment.

a H3K4me3 and Morc3 ChIP-seq coverage at promoters

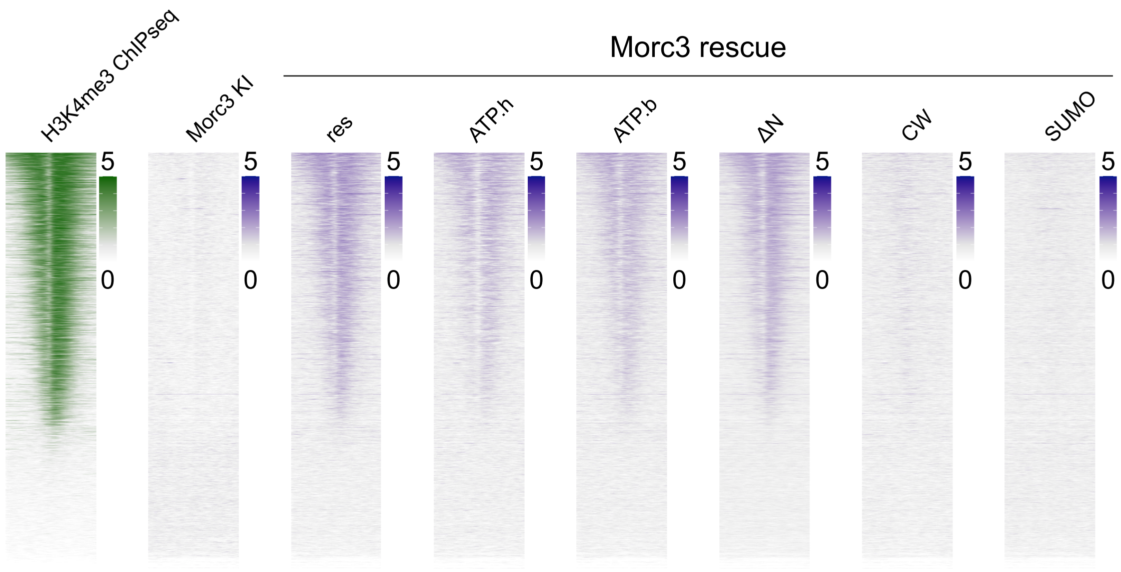

b H3K4me3 ChIP-seq coverage at promoters in wt vs Morc3 ko

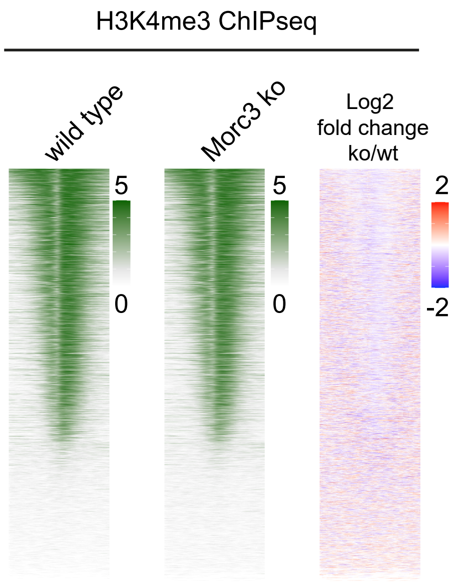

Figure S9. Morc3 can associate with H3K4me3 promoters. a Read-density heat maps showing the normalized coverage of H3K4me3 and Morc3 wild type and mutant proteins on promoters. Wild type Morc3 from the 3xFLAG knock-in allele could not be detected on promoters, whereas the Morc3 from wild type rescue cells could be detected. Morc3 mutant proteins display promoter association with exception of the CW and SUMO mutants. **b** Read-density heat map showing the normalized coverage of H3K4me3 in wt and Morc3 ko cells. No pronounced change in H3K4me3 signal was observed.

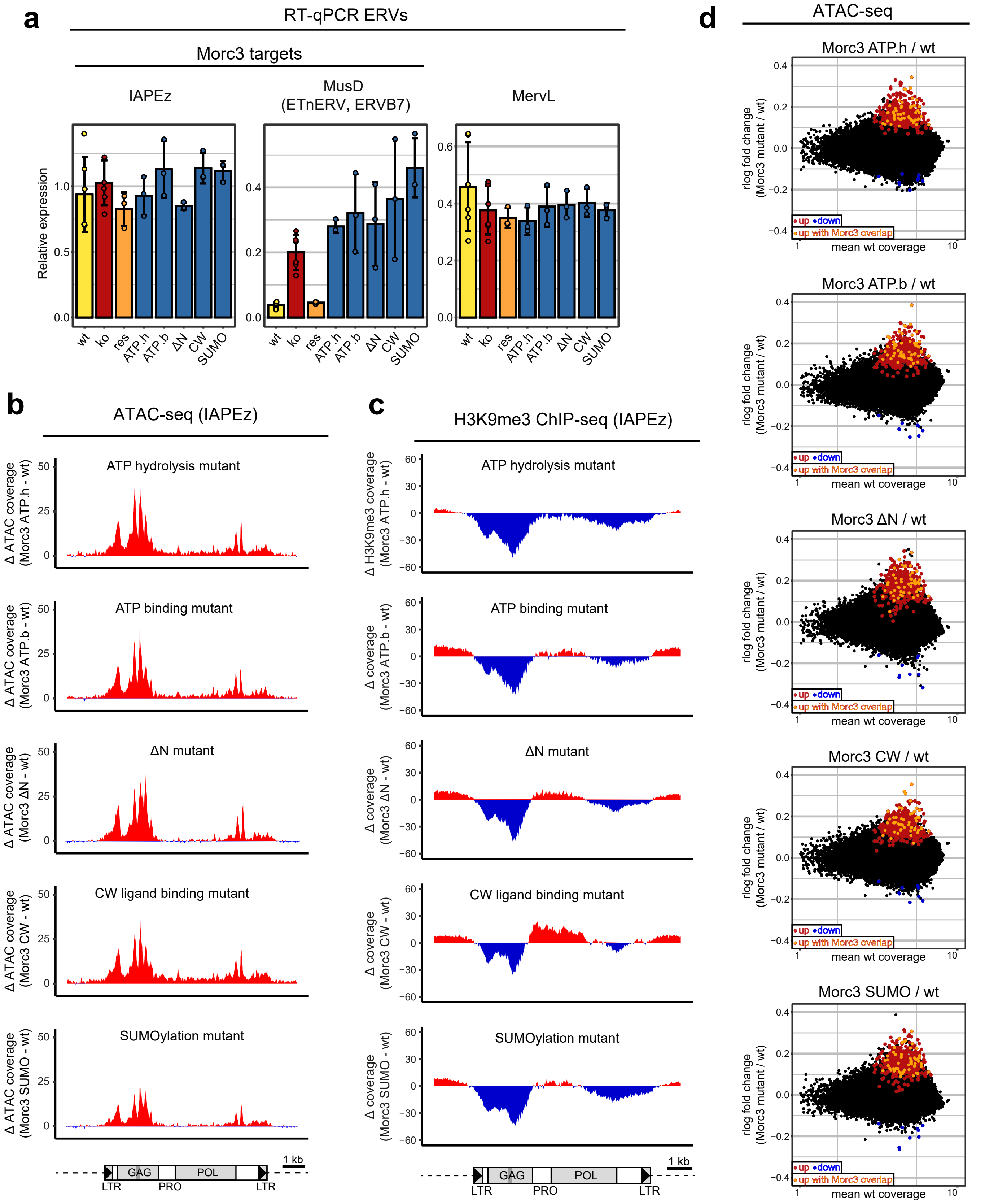

Figure S10. Morc3 mutants fail to rescue Morc3-dependent chromatin changes. a RT-qPCR analysis of ERV expression in Morc3 mutant rescue ES cells. Bar graph depicts mean relative expression to control genes (*Actin* and *Hprt*). Error bars indicate standard deviation of replicate experiments (n=3). **b** Increased chromatin accessibility on IAPEz elements cannot be rescued by Morc3 mutant proteins. Plots display the difference in cumulative ATAC-seq coverage between Morc3 mutant and wild type ES cells. The position of the SHIN sequence is indicated as gray bar.

Continued legend Figure S10. c Decreased H3K9me3 levels on IAPEz elements cannot be rescued by Morc3 mutant proteins. Plots display the difference in cumulative H3K9me3 ChIP-seq coverage between Morc3 mutant and wild type ES cells. The position of the SHIN sequence is indicated as a gray bar. **d** Dot plot showing average coverage vs. log2-fold change of ATAC peaks in wild type vs. Morc3 mutant rescue ES cells. Colored dots indicate ATAC peaks with significantly increased (red dots) or decreased (blue dots) coverage in Morc3 ko ES cells (Figure 4c), peaks with significantly increased ATAC coverage and overlapping with Morc3 peaks are marked in orange. ATAC coverage of these peaks cannot be rescued in all tested Morc3 mutant rescue ES cells.

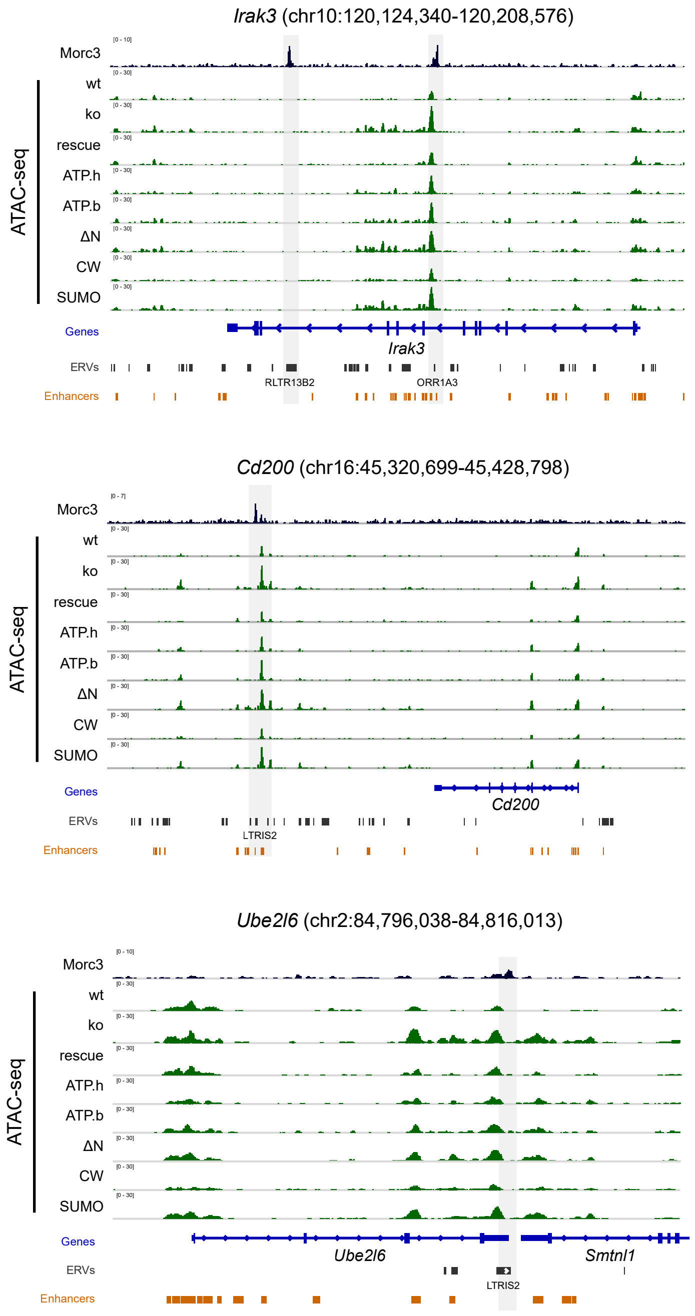

Figure S11. Morc3 mutant proteins fail to rescue increased chromatin accessibility on Morc3 targets. Genome browser view of chromatin accessibility on Morc3 target genes (*Irak3, Cd200, Ube2l6*). Positions of Morc3 peaks are indicated by gray boxes. wt – wild type, ko – Morc3 ko, rescue – Morc3 wild type rescue, ATP.h – ATP hydrolysis mutant, ATP.b – ATP binding mutant, ΔN – Dimerization mutant, CW – CW mutant, SUMO – SUMOylation mutant.

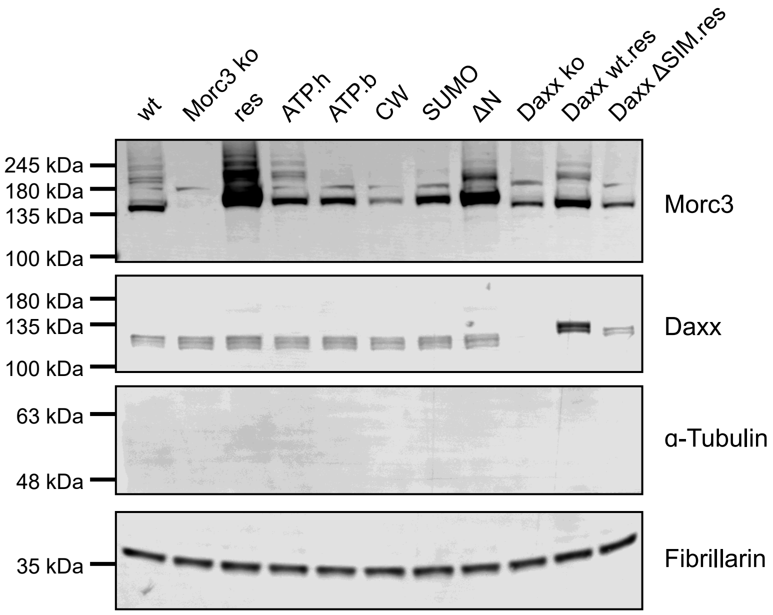

Figure S12. Nuclear Daxx levels are not changed in Morc3 mutant rescue cell lines. Western blot analysis of nuclear extracts from wild type, Morc3 ko, Morc3 rescue, Daxx ko and Daxx rescue cell lines using antibodies against Morc3, Daxx, alpha-Tubulin (cytoplasmic control) and Fibrillarin (loading control). Nuclear Daxx levels are similar in all Morc3 mutant cell lines. Detection on the same blot with the LI-COR system. Uncropped blot in Source Data.

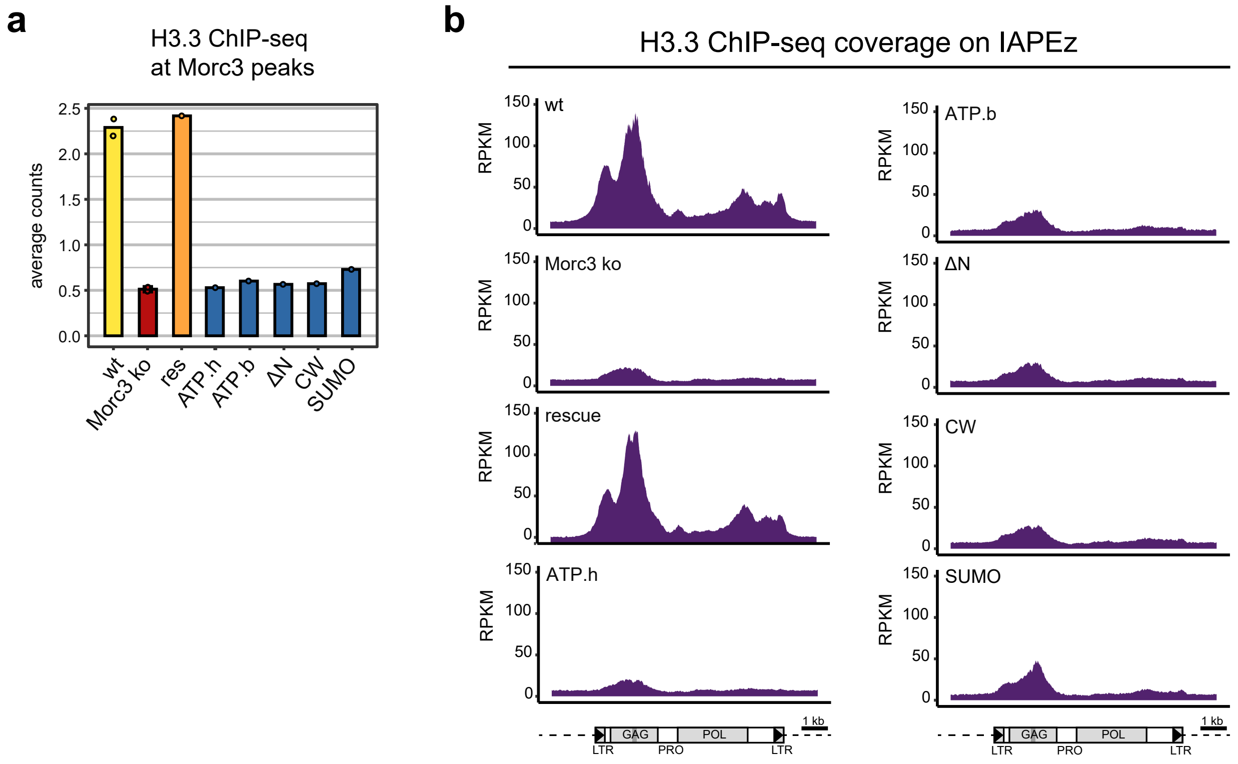

Figure S13. Histone H3.3 ChIPseq analysis. a Histone H3.3 coverage on Morc3 peaks. Bar graph depicts average normalized reads over all Morc3 peak centers (100 bp bin). Bar of wt and ko shows mean of replicate experiments (n=2). ChIP-seq of rescue cells was not replicated (n=1). **b** Cumulative coverage plot of histone H3.3 on IAPEz elements. In wt and rescue cells, prominent enrichment is detected over the 5'UTR and the GAG region. The position of the SHIN sequence is indicated as dark gray bar. In all Morc3 mutant rescue cell lines histone H3.3 coverage is strongly reduced on IAPEz elements.

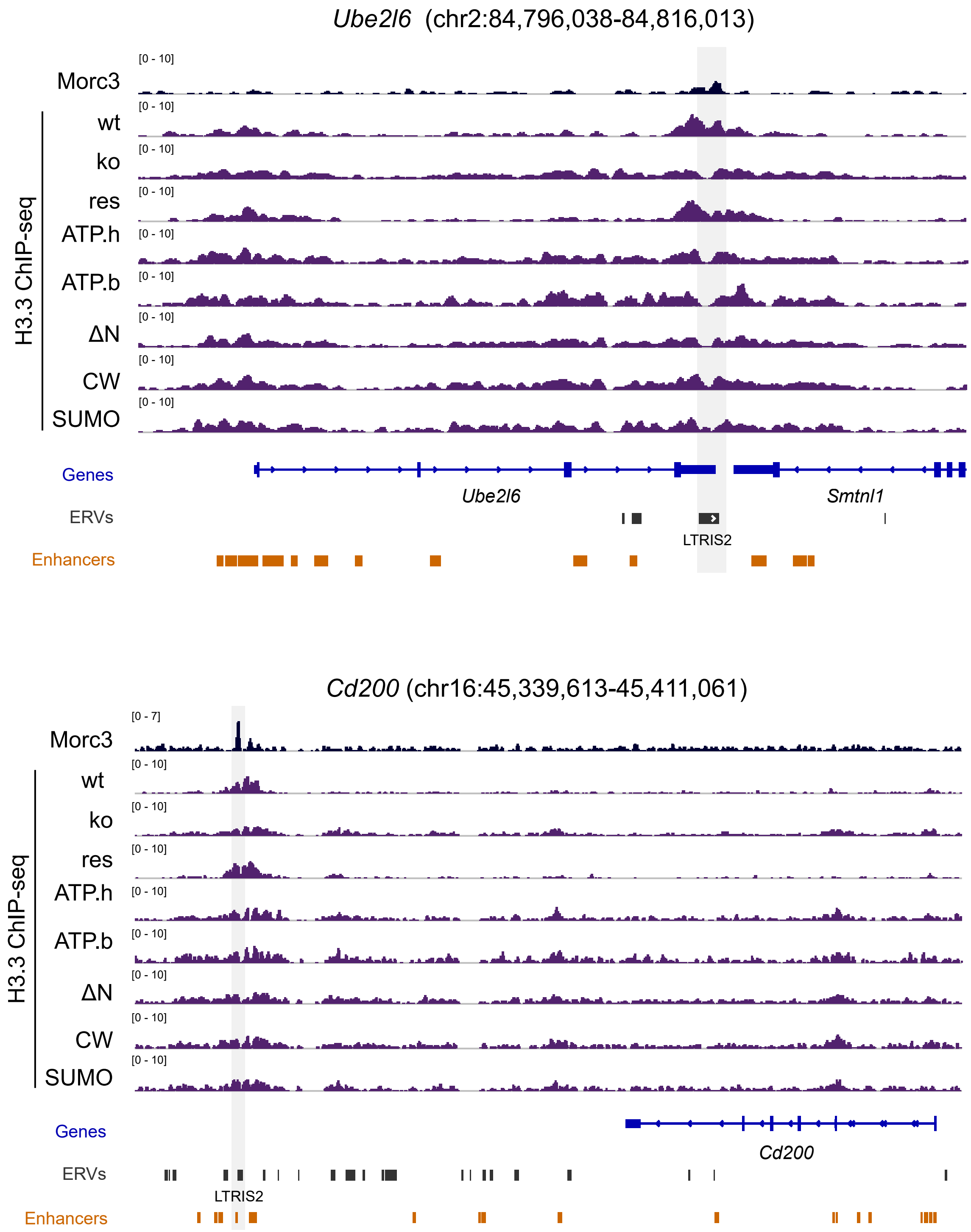

Figure S14. Morc3 mutant proteins fail to rescue histone H3.3 enrichment on Morc3 target genes. Genome browser views of histone H3.3 enrichment on additional Morc3 target genes (*Ube2l6*, *Cd200*), complementary to Figure 7b. Positions of Morc3 peaks are indicated by gray boxes. wt – wild type, ko – Morc3 ko, rescue – Morc3 wild type rescue, ATP.h – ATP hydrolysis mutant, ATP.b – ATP binding mutant, ΔN – Dimerization mutant, CW – CW mutant, SUMO – SUMOylation mutant.

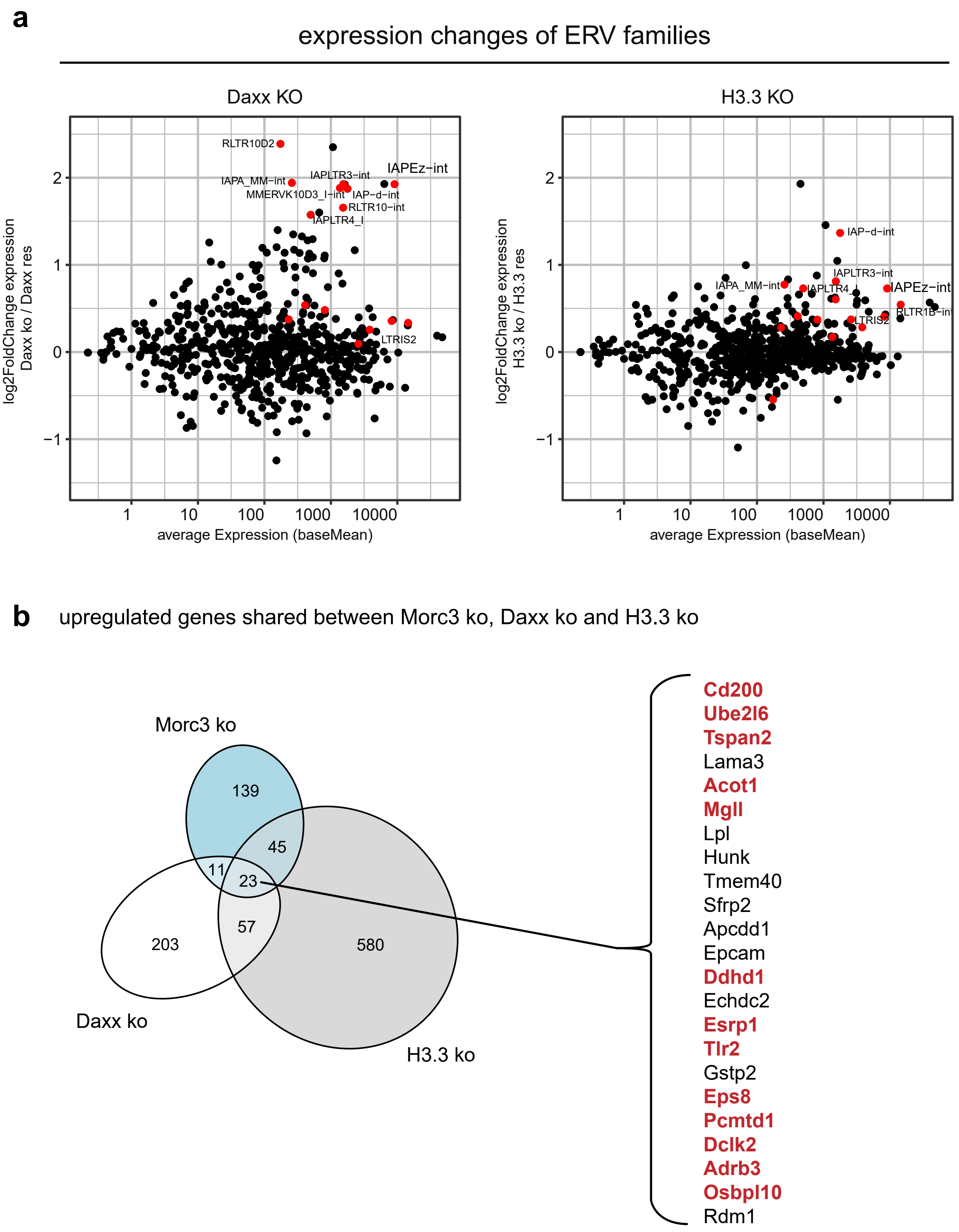

Figure S15. Regulation of Morc3 target ERVs and genes in Daxx and H3.3 ko cells. a Dot plot showing average expression vs. log2-fold change of ERV families in rescue vs. Daxx knock-out (left panel) and rescue vs. H3.3 knock-out (right panel) cells. Colored dots indicate ERVs with significantly changed expression in Morc3 ko (Figure 3f). Positions of relevant ERV families are indicated. Expression data are from Hoelper et al.[2] **b** Venn diagram showing overlap of significantly (adjusted p-value < 0.05, by DESeq2) up-regulated genes shared between Daxx ko, H3.3 ko and Morc3 ko. Morc3 target genes (Supplementary Data 5) are marked in red in the list of overlapping genes.

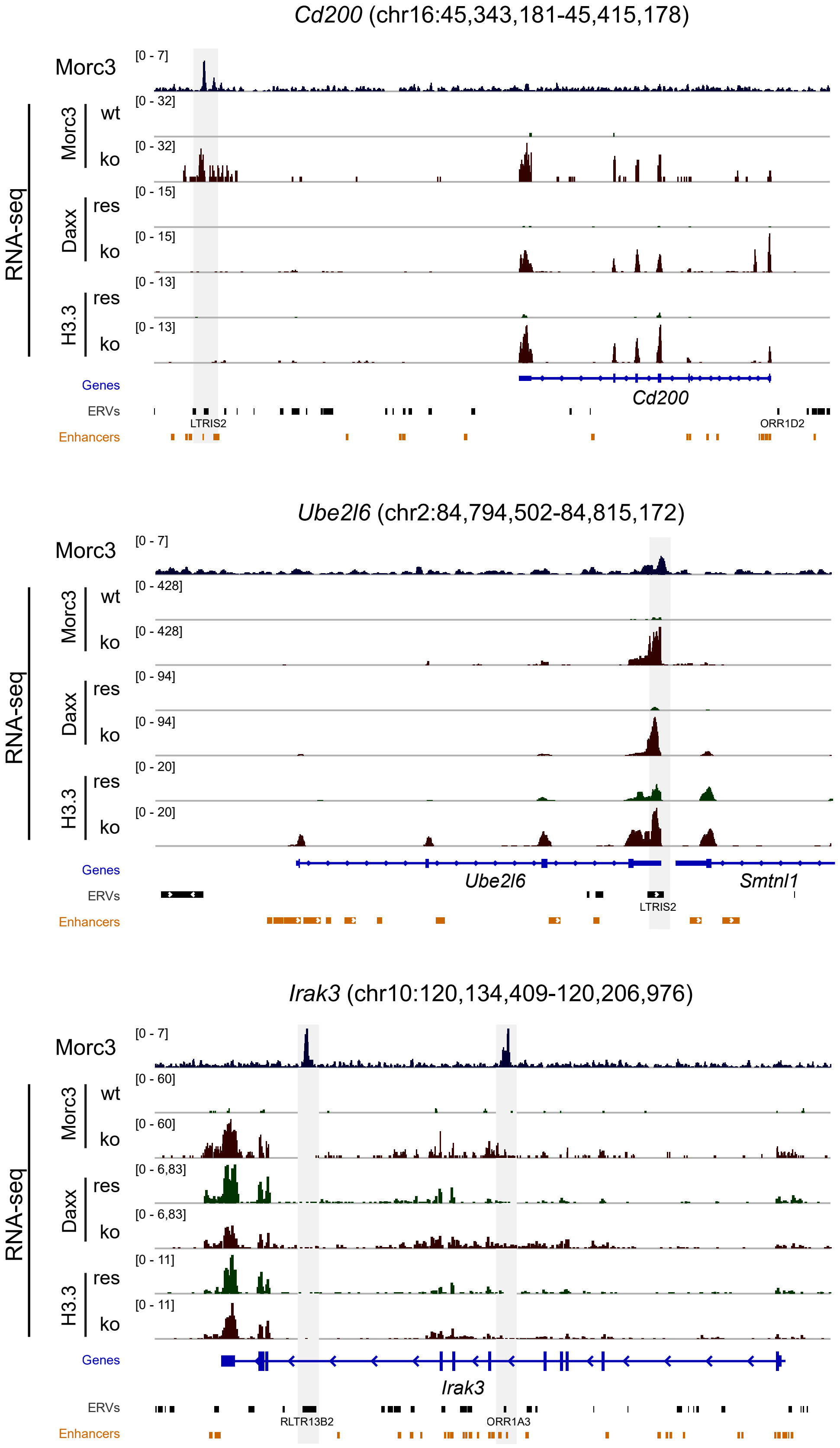

Figure S16. Morc3 target genes are similarly regulated in Daxx, H3.3 and Morc3 ko cells. Genome browser view of RNA-seq coverage on Morc3 target genes (*Cd200, Ube2l6, Irak3*). Positions of Morc3 peaks are indicated by gray boxes.

Table S1. Plasmids. List of all plasmids used for lentiviral packaging, sgRNA knock-out, SHIN reporter assays, and generation of cell lines with Daxx rescue, Morc3 knock-in, Morc3 knock-out and Morc3 rescue.

ID	Name	Purpose
#183	psPax2	packaging lentivirus
#811	pLP-ecoenv	packaging lentivirus
#1348	pLenti6-sgRNA-ccdB	backbone for lentiviral sgRNA Plasmids
#940	pLenti6-EFEGT-neo	SHIN initiation assay
#1074	pLenti6-EFEGT-neo/GAG2.22	SHIN initiation assay
#1272	pLFIP-3xFLAG-Daxx_FL	3xFLAG-Daxx expression for rescue
#1273	pLFIP-3xFLAG-Daxx_delta_SIM	3xFLAG-Daxx_delta_SIM expression for mutant rescue
#1523	pX330-Morc3_knock_in	CRISPR cut for Morc3-3xFLAG knock in
#1529	pBlu2SKP_Morc3_3xflag_KI	Template for homologous repair of Morc3 CRISPR cut including 3xFLAG
#1500	pX330-Morc3_del1	Generation of Morc3 KO cell lines
#1501	pX330-Morc3_del2	Generation of Morc3 KO cell lines
#1704	pCMV-hyPBase	PiggyBac (PB) Transposase
#1721	PB_hPGK_Morc3_T2A_Puro	Morc3 expression for rescue
#1725	PB_hPGK_Morc3_E35A_T2A_Puro	Morc3_E35A expression for mutant rescue
#1727	PB_hPGK_Morc3_G101A_T2A_Puro	Morc3_G101A expression for mutant rescue
#1728	PB_hPGK_Morc3_W419A_T2A_Puro	Morc3_W419A expression for mutant rescue
#1759	PB_hPGK_Morc3_3xFLAG_T2A_Puro	Morc3_3xFLAG expression for rescue
#1760	PB_hPGK_Morc3_E35A_3xFLAG_T2A_Puro	Morc3_E35A_3xFLAG expression for mutant rescue
#1762	PB_hPGK_Morc3_G101A_3xFLAG_T2A_Puro	Morc3_G101A_3xFLAG expression for mutant rescue
#1763	PB_hPGK_Morc3_W419A_3xFLAG_T2A_Puro	Morc3_W419A_3xFLAG expression for mutant rescue
#1814	PB_hPGK_Morc3_SUMOylation_3xFLAG_T2A_Puro	Morc3_SUMOylation_3xFLAG expression for mutant rescue
#1817	PB_hPGK_Morc3_deltaN_3xFLAG_T2A_Puro	Morc3_deltaN_3xFLAG expression for mutant rescue

Table S2. sgRNA oligonucleotides. Sequences used for cloning of sgRNAs in lentiviral vectors for knock-out of indicated targets in SHIN silencing experiments.

Target name	Forward sequence 5' - 3'	Reverse sequence 5' - 3'	ID fwd	ID rev
sgSetdb1	CACC GCTGAGCTGCAGCAGGCGG	AAAC CCGCCTGCTGCAGCTCAGC	GS3110	GS3111
sgAtf7ip	CACC GCCACTACAGTACAGGCTAC	AAAC GTAGCCTGTACTGTAGTGGC	GS2769	GS2770
sgDnmt1	CACC GCTTCGTGAAGTGAGCCGTGA	AAAC TCACGGCTCACTTCACGAAGC	GS3526	GS3527
sgUhrf1	CACC GTCACAGTGCGAGCACGAGCA	AAAC TGCTCGTGCTCGCACTGTGAC	GS3528	GS3529
sgDaxx	CACC GTACAATGATGCTGTCAT	AAAC ATGACAGCATCATTGTAC	GS3108	GS3109
sgTrim28	CACC GCCGCGTCGTCCCCTGCG	AAAC CGCAGGGGACGACGCGGC	GS3112	GS3113
sgAtrx	CACC GACAACTCCTTTCGACCA	AAAC TGGTCGAAAGGAGTTGTC	GS3106	GS3107
sgMorc3	CACC GTGAATGCTAAACAGATC	AAAC GATCTGTTTAGCATTCAC	GS3439	GS3440
sgZfp445	CACC GTTCCATGTGAAGTCTCCAAG	AAAC CTTGGAGACTTCACATGGAAC	GS3536	GS3537
sgZfp54	CACC GAAATCCTATGCCTACCGTAC	AAAC GTACGGTAGGCATAGGATTTC	GS3534	GS3535
sgZfp90	CACC GCACGTCCCTCACGCAACATG	AAAC CATGTTGCGTGAGGGACGTGC	GS3532	GS3533
sgGtf2ird1	CACC GCATTGTGTACCGCCACGC	AAAC GCGTGGCGGTACACAATGC	GS3437	GS3438
sgZfp606	CACC GATCAACCCATGGCCCTCC	AAAC GGAGGGCCATGGGTTGATC	GS3171	GS3200
sgPrdm5	CACC GAAAATTTGGACCCTTCGC	AAAC GCGAAGGGTCCAAATTTTC	GS3435	GS3436
sgPogK	CACC GCCTCGGAATGCCCGCCGCA	AAAC TGCGGCGGGCATTCCGAGGC	GS3538	GS3539
sgZfp943	CACC GTAGCTCTGTAAGATGTACTT	AAAC AAGTACATCTTACAGAGCTAC	GS3540	GS3541
sgSmarcal1	CACC GACAACTTCTCAGCTCGAC	AAAC GTCGAGCTGAGAAGTTGTC	GS3433	GS3434
sgTrim24	CACC GCCGCCGCTCTCCGAGTC	AAAC GACTCGGAGAGCGGCGGC	GS3441	GS3442
sgZfp599	CACC GCATTTGAAGACGTAGCCG	AAAC CGGCTACGTCTTCAAATGC	GS3443	GS3444
sgPhf19	CACC GCCACCAGTCCTAACAAG	AAAC CTTGTTAGGACTGGTGGC	GS3445	GS3446
sgDsn1	CACC GATGACGCAAGGCGTTTCAG	AAAC CTGAAACGCCTTGCGTCATC	GS3530	GS3531
sgZfp3	CACC GAAGACTCCAAGCCACAT	AAAC ATGTGGCTTGGAGTCTTC	GS3431	GS3432
sgNeg	CACC GTTTGGCTCTACAAAGGC	AAAC GCCTTTGTAGAGCCAAAC	GS3126	GS3127
sgDek	CACC GGGTTCCTTCTCGGATGA	AAAC TCATCCGAGAAGGAACCC	GS3120	GS3121
sgKdm5b	CACC GACACCCGAGTGTATAGTC	AAAC GACTATACACTCGGGTGTC	GS4117	GS4118
sgEhmt1	CACC GCAAGGTGTCTCTTTGTTCT	AAAC AGAACAAAGAGACACCTTGC	GS4115	GS4116
sgMorc2a_del6	CACC GCCATACTGCCCAATTTGAG	AAAC CTCAAATTGGGCAGTATGGC	GS4370	GS4371
sgMorc2a_del7	CACC GCTCAATGCGGATTGGGA	AAAC TCCCAATCCGCATTGAGC	GS4372	GS4373
sgMorc2a_del8	CACC GCACCTGGAATGCTCGAACAC	AAAC GTGTTCGAGCATTCCAGGTGC	GS4374	GS4375
sgCbx1	CACC GCGTTGCGTCGGGCGGTCCGA	AAAC TCGGACCGCCCGACGCAACGC	GS4140	GS4141
sgCbx3	CACC GTGGCGCCGATTATTCGTTC	AAAC GAACGAATAATCGGCGCCAC	GS4142	GS4143
sgCbx5	CACC GTTGGACAGGCGCATGGTTAA	AAAC TTAACCATGCGCCTGTCCAAC	GS4144	GS4145
sgEzh2	CACC GTTCTTTAGACTCTTCCCTG	AAAC CAGGGAAGAGTCTAAAGAAC	GS4113	GS4114
sgEed	CACC GCGTATTTGTGGGCGTGTC	AAAC GACACGCCCACAAATACGC	GS3979	GS3980

Table S3. Cell lines. Summary of all cell lines used and generated.

ID	Description	Background	Source
wt26	wild type mouse ES cell line	-	Peters et al. [3]
A9	mouse ES cell line (C57/BL6J 3 129/Sv ES)	-	Nakashima et al. [4]
T86	Stable integration by RMCE at HA36 of doxycylin inducible SHIN reporter, expressing rtTA	HA36	Sadic et al. [5]
T37	HeLa cell expressing the mouse lentiviral receptor Slc7a1 (mCAT)	HeLa	Sadic et al. [5]
293T	Commercial human embryonic kidney cells for virus packaging	HEK	Invitrogen
T90	T86 expressing Cas9	T86	This study
T89	Wt cells expressing Cas9	wt26	This study
KO27-1	Morc3 KO (single clone)	wt26	This study
KO27-2	Morc3 KO (single clone)	wt26	This study
KO27-3	Morc3 KO (single clone)	wt26	This study
K9-G1	Setdb1-3xFLAG KI (single clone)	A9	Schotta lab
K14-E8	Morc3-3xFLAG KI (single clone)	wt26	This study
T90-B1	T90 (single clone)	T90	This study
T90-B2	T90 (single clone)	T90	This study
T90-D1	T90 (single clone)	T90	This study
KO43-4	Morc3 KO (single clone)	T90	This study
KO43-7	Morc3 KO (single clone)	T90	This study
KO43-8	Morc3 KO (single clone)	T90	This study
T131-3C	wt- Morc3 rescue (by PiggyBac, single clone)	KO27-2	This study
T131-3D	wt- Morc3 rescue (by PiggyBac, single clone)	KO27-2	This study
T131-3M	wt- Morc3 rescue (by PiggyBac, single clone)	KO27-2	This study
T141-5	Morc3_E35A-rescue ATP hydrolysis mutant (by PiggyBac, single clone)	KO27-2	This study
T141-7	Morc3_E35A-rescue ATP hydrolysis mutant (by PiggyBac, single clone)	KO27-2	This study
T141-9	Morc3_E35A-rescue ATP hydrolysis mutant (by PiggyBac, single clone)	KO27-2	This study
T142-4	Morc3_G101A-rescue ATP binding mutant (by PiggyBac, single clone)	KO27-2	This study
T142-5	Morc3_G101A-rescue ATP binding mutant (by PiggyBac, single clone)	KO27-2	This study
T142-11	Morc3_G101A-rescue ATP binding mutant (by PiggyBac, single clone)	KO27-2	This study
T143-2	Morc3_W419A-rescue CW domain mutant (by PiggyBac, single clone)	KO27-2	This study
T143-3	Morc3_W419A-rescue CW domain mutant (by PiggyBac, single clone)	KO27-2	This study
T143-5	Morc3_W419A-rescue CW domain mutant (by PiggyBac, single clone)	KO27-2	This study
T145-5	Morc3_3xFLAG-rescue wt (by PiggyBac, single clone)	KO27-2	This study
T146-5	Morc3_E35A_3xFLAG rescue ATP hydrolysis mutant (by PiggyBac, single clone)	KO27-2	This study
T148-3	Morc3_G101A_3xFLAG-rescue ATP binding mutant (by PiggyBac, single clone)	KO27-2	This study
T149-3	Morc3_W419A_3xFLAG-rescue CW domain mutant (by PiggyBac, single clone)	KO27-2	This study
T181-6D	Morc3_SUMOylation_3xFLAG-rescue SUMOylation mutant (by PiggyBac, single clone)	KO27-2	This study
T181-7J	Morc3_SUMOylation_3xFLAG-rescue SUMOylation mutant (by PiggyBac, single clone)	KO27-2	This study
T181-15	Morc3_SUMOylation_3xFLAG-rescue SUMOylation mutant (by PiggyBac, single clone)	KO27-2	This study
T185-5	Morc3_deltaN_3xFLAG-rescue deltaNmutant (by PiggyBac, single clone)	KO27-2	This study
T185-7	Morc3_deltaN_3xFLAG-rescue deltaNmutant (by PiggyBac, single clone)	KO27-2	This study
T185-12	Morc3_deltaN_3xFLAG-rescue deltaNmutant (by PiggyBac, single clone)	KO27-2	This study
KO2-3	Daxx KO (single clone)	wt26	Sadic et al. [5]
T163-8	3xFLAG-Daxx full length rescue (single clone)	KO2-3	This study
T163-4	3xFLAG-Daxx full length rescue (single clone)	KO2-3	This study
T163-9	3xFLAG-Daxx full length rescue (single clone)	KO2-3	This study
T164-3	3xFLAG-Daxx delta SIM rescue (single clone)	KO2-3	This study
T164-7	3xFLAG-Daxx delta SIM rescue (single clone)	KO2-3	This study
T164-8	3xFLAG-Daxx delta SIM rescue (single clone)	KO2-3	This study

Table S4. Antibodies. Antibodies used for western blot, Chromatin immunoprecipitation and Immunofluorescence.

ID	Name	Source/ Company	Cat#/ Clone	Purpose	Dilution in Westernblot
#67	NP95	Leonhardt Lab	clone 8H3	Westernblot loading control	1:5
#075	Nanog	Biomol, Bethyl	A300-397A	Westernblot loading control	1:1000
#232	FLAG M2	Sigma	F1804	Westernblot, ChIP-seq, ChIP-MS	1:1000
#249	H3K9me3	Active Motif	39161	ChIP-seq	-
#251	LaminB1	Active Motif	39095	Westernblot loading control	1:1000
#259	H3K4me3	Diagenode	CS-003-100	ChIP-seq	-
#265	H3K27ac	Diagenode	pAB-174-050	ChIP-seq	-
#285	Morc3	Rockland	100-401-N96S	Westernblot, Immunofluorescence	1:1000
#304	Fibrillarin	Santa Cruz	sc-166021	Westernblot loading control	1:100
#316	Daxx(H-7)	Santa Cruz	sc-8043	Westernblot	1:500
#318	Histone H3.3	Millipore	#09-838	ChIP-seq	-

Table S5. RT-qPCR oligonucleotides. Primers used for expression analysis by RT-qPCR.

Name/ Target	ID	Direction	Sequence (5' to 3')	Use	Property	References for published RT-qPCR primers
Actin	GS278.1	fwd	GGTCATCACTATTGGCAACG	Fig3C, Fig5D, FigS08B, FigS10A used as reference	Housekeeping used for normalization	Sadic et al. [5]
	GS278.2	rev	TCCATACCCAAGAAGGAAGG			
Hprt1	GS279.1	fwd	ATGAGCGCAAGTTGAATCTG	Fig3C, Fig5D, FigS08B, FigS10A used as reference	Housekeeping used for normalization	–
	GS279.2	rev	CAGATGGCCACAGGACTAGA			
Tbc1d13	GS4655	fwd	GGCTCTATTCGCAGTTCCTG	Fig3C	Morc3 target gene	–
	GS4656	rev	ATGTGTTCCACCTGCTGTCA			
Eps8	GS4643	fwd	CCAGACCTTCACCTTTTCCA	Fig3C	Morc3 target gene	–
	GS4644	rev	GCCTCTTCTGTTTCCCACCT			
Irak3	GS4633	fwd	ACCCTGTCCTCGGAACTTCT	Fig3C, Fig5D	Morc3 target gene	–
	GS4634	rev	TACAAGCTAGGCTGGGTGCT			
Ube2l6	GS4639	fwd	GAATCTGGAAGAGCCTGTGC	Fig3C, Fig5D	Morc3 target gene	–
	GS4640	rev	CGGTCCACTCCGAATTTAAG			
Cd200	GS4651	fwd	CCTGGGGAATGTGATTGACT	Fig3C, Fig5D	Morc3 target gene	–
	GS4652	rev	CTCCTGATTTCGGTGACGTT			
Morc3	GS3870	fwd	TAAAGGCGGAGCACGTTG	FigS08B	Morc3 expression	–
	GS3871	rev	TTCCAGAATTGCTGCTAGGC			
IAPEz	GS4250	fwd	CCTGTGCAGCGGTATAAGGT	FigS10A	Morc3 target ERV	–
	GS4251	rev	CCAAACGTATCCCCAGCTAA			
MusD	GS3453	fwd	GATTGGTGGAAGTTTAGCTAGCAT	FigS10A	Morc3 target ERV	Rowe et al. [6]
	GS3454	rev	TAGCATTCTCATAAGCCAATTGCAT			
Merv-L	GS3455	fwd	CTTCCATTCACAGCTGCGACTG	FigS10A	non-target ERV, negative control	Maksakova et al. [7]
	GS3456	rev	CTAGAACCACTCCTGGTACCAAC			

Table S6. Oligonucleotides for cloning.

Cloning of sgRNA sequence for targeting Morc3 knock-out and knock-in

Name/ Target	ID	Direction	Sequence (5' to 3')	Use	Property
Morc3_del.1	GS3703	fwd	CACC GCAAGTCAGGTTCTATGCGAC	CrispR	Generation of Morc3 KO cells
	GS3704	rev	AAAC GTCGCATAGAACCTGACTTGC		
Morc3_del.2	GS3705	fwd	CACC GCTCCGCCTTTATGACTTCC	CrispR	Generation of Morc3 KO cells
	GS3706	rev	AAAC GGAAGTCATAAAGGCGGAGC		
Morc3_knock_in	GS3741	fwd	CACC GATGAGATTCTAGGACAAG	CrispR	Generation of Morc3 KI cells
	GS3742	rev	AAAC CTTGTCCTAGAATCTCATC		

Cloning of Morc3-3xFLAG Knock in by Gibson Assembly

Name/ Target	ID	Direction	Sequence (5' to 3')	Use	Property
Morc3_5'HR	GS3743	fwd	gtcaacctgacaaagtttactaggac	Knock-in	Amplification of 5' homology arm for KI
	GS3744	rev	agtactgctgatctcgctcatctgctccaCTActtgtcctag		
Morc3_3'HR	GS3745	fwd	ggcagcGACTACAAAGACCATGACGGTGATTATAAAGATCA TGACATCGACTACAAGGATGACGATGACAAGTAAGAATTC gtgcagacgtctggtgatgg	Knock-in	Amplification of 3' homology arm for KI including 3xFLAG Tag
	GS3746	rev	cccaggcccatttatgatta		
Morc3_GA_vector-5'HR	GS3747	fwd	gatatcgaattcctgcaGTCAACCTGACAAAGTTTACTAG	Knock-in	
Morc3_GA_3'HR- 5'HR	GS3748	rev	tcgctgccAGTACTGCTGATCTCGCTC	Knock-in	
Morc3_GA_5'HR-3'HR	GS3749	fwd	gcagtactGGCAGCGACTACAAAGAC	Knock-in	
Morc3_GA_vector-3'HR	GS3750	rev	atcccccgggctgcaCCCAGGCCCATTTATGATTAAC	Knock-in	
Check_GA_Morc3	GS3751	fwd	gctgctcacttcctgactcc	Knock-in	

Cloning of Morc3 rescue by Gibson Assembly

Name/ Target	ID	Direction	Sequence (5' to 3')	Use	Property
Morc3_cDNA	GS4247	fwd	ATGGCGGCGCAGCCACCC	cloning	Amplification of Morc3 cDNA (without the STOP codon)
	GS4248	rev	AGTACTGCTGATCTCGCTCATCTGCTCCAC	cloning	
Morc3_3xFLAG	GS4287	fwd	ggcagcGACTACAAAGACCATGACGGTGATTAT AAAGATCATGACATCGACTACAAGGATGACGA TGACAAGGGAGGAGAGGGAAGAGGAAG	Gibson Assembly cloning	Introduction of 3xFLAG Tag to Morc3 rescue plasmids
	GS4288	rev	gtctttgtagtcgctgccAGTACTGCTGATCTCGCTCAT C	Gibson Assembly cloning	
Morc3_W419A	GS4279	fwd	gtctaaag**gcc**CGAAAGTTACCAGATGGGATAG	Gibson Assembly cloning	Creation of W419A point mutation in Morc3 rescue plasmids
	GS4280	rev	ctttcg**ggc**CTTTAGACAAGCGTCACATTGAAC	Gibson Assembly cloning	
Morc3_E35A	GS4281	fwd	cagttgct**gcc**TTAATAGATAATGCTTATGATCCTG ATGTG	Gibson Assembly cloning	Creation of E35A point mutation in Morc3 rescue plasmids
	GS4282	rev	ctattaa**ggc**AGCAACTGCACTGAACGG	Gibson Assembly cloning	
Morc3_G101A	GS4285	fwd	gattgtac**gcc**AATGGCTTCAAGTCAGGTTCTATG	Gibson Assembly cloning	Creation of G101A point mutation in Morc3 rescue plasmids
	GS4286	rev	gccatt**ggc**GTACAATCCAACTGGGACATGAC	Gibson Assembly cloning	
Morc3_SUMO_ insert	GS4623	fwd	AAATCAGATATTGAAGTCCGGTCAG	Gibson Assembly cloning	Insertion of artificial fragment containing SUMOylation mutations in Morc3 rescue plasmids
	GS4624	rev	TCTCAGTGCAGGAAGCCTG	Gibson Assembly cloning	
Morc3_deltaN	GS4725	fwd	TAGAATTTGTATGTAAAAACTT CATCTTGGATCCGATATCCC	Gibson Assembly cloning	Creation of delta N (aa2-16) mutation in Morc3 rescue plasmids
	GS4726	rev	AAGTTTTTACATACAAATTCTACTAGTCAT	Gibson Assembly cloning	

Primer for PCR or sanger sequencing confirmation of cloned plasmids

Name/ Target	ID	Direction	Sequence (5' to 3')	Use
Morc3_del_check_fwd	GS3707	fwd	gttggattgtacgggaatgg	PCR
Morc3_del_check_rev	GS3708	rev	tttggacgagtgaggtgttg	PCR
Morc3_KI_check_out5'HA	GS3868	fwd	gctgctcacttcctgactcc	PCR
Morc3_KI_check_in3'HA	GS3869	rev	gctattacgctgctcaaccc	PCR
Morc3_seq.1	GS3698	f	ttatgatcctgatgtgaatg	sequencing
Morc3_seq.2	GS3699	f	ggaatctcagaagttacaaa	sequencing
Morc3_seq.3	GS3700	f	aggagaaaagctgaatgatt	sequencing
Morc3_seq.4	GS3701	f	tgagaacagtagcatgaagcggaaa	sequencing
Morc3_seq.5	GS3702	f	agtcacacaagaaagagatg	sequencing

Table S6. Oligonucleotides for library preparation of sgRNA screen

CrispR library NGS PCR

Name	ID	Direction	Sequence (5' to 3')	Use
CrispR NGS PCR1	GS3367	fwd	aatggactatcatatgcttaccgtaacttgaaagtatttcg	library preparation of CRISPR screen
CrispR NGS PCR1	GS3368	rev	ctttagtttgtatgtctgttgctattatgtctactattctttcc	library preparation of CRISPR screen
CrispR NGS PCR2	GS3369	fwd	AATGATACGGCGACCACCGAGATCTACACTCTTTCCCTACACGACGCT CTTCCGATC*Tcttgtggaaaggacgaaacaccg	library preparation of CRISPR screen
CrispR NGS PCR2 #11	GS3370	rev	CAAGCAGAAGACGGCATACGAGAT<u>GTAGCC</u>GTGACTGGAGTTCAGAC GTGTGCTCTTCCGATC*Tctactattctttcccctgcactgt	library preparation of CRISPR screen
CrispR NGS PCR2 #9	GS3371	rev	CAAGCAGAAGACGGCATACGAGAT<u>CTGATC</u>GTGACTGGAGTTCAGAC GTGTGCTCTTCCGATC*Tctactattctttcccctgcactgt	library preparation of CRISPR screen

Table S7. Primers used for ATAC-seq from Buenrostro, et al. [8]

Name	ID	Direction	Sequence (5' to 3')
Ad1_noMX	Ad1	fwd	AATGATACGGCGACCACCGAGATCTACACTCGTCGGCAGCGTCAGATGTG
Ad2.1_TAAGGCGA	Ad2.1	rev	CAAGCAGAAGACGGCATACGAGATTCGCCTTAGTCTCGTGGGCTCGGAGATGT
Ad2.2_CGTACTAG	Ad2.2	rev	CAAGCAGAAGACGGCATACGAGATCTAGTACGGTCTCGTGGGCTCGGAGATGT
Ad2.3_AGGCAGAA	Ad2.3	rev	CAAGCAGAAGACGGCATACGAGATTTCTGCCTGTCTCGTGGGCTCGGAGATGT
Ad2.4_TCCTGAGC	Ad2.4	rev	CAAGCAGAAGACGGCATACGAGATGCTCAGGAGTCTCGTGGGCTCGGAGATGT
Ad2.5_GGACTCCT	Ad2.5	rev	CAAGCAGAAGACGGCATACGAGATAGGAGTCCGTCTCGTGGGCTCGGAGATGT
Ad2.6_TAGGCATG	Ad2.6	rev	CAAGCAGAAGACGGCATACGAGATCATGCCTAGTCTCGTGGGCTCGGAGATGT
Ad2.7_CTCTCTAC	Ad2.7	rev	CAAGCAGAAGACGGCATACGAGATGTAGAGAGGTCTCGTGGGCTCGGAGATGT
Ad2.8_CAGAGAGG	Ad2.8	rev	CAAGCAGAAGACGGCATACGAGATCCTCTCTGGTCTCGTGGGCTCGGAGATGT
Ad2.9_GCTACGCT	Ad2.9	rev	CAAGCAGAAGACGGCATACGAGATAGCGTAGCGTCTCGTGGGCTCGGAGATGT
Ad2.10_CGAGGCTG	Ad2.10	rev	CAAGCAGAAGACGGCATACGAGATCAGCCTCGGTCTCGTGGGCTCGGAGATGT
Ad2.11_AAGAGGCA	Ad2.11	rev	CAAGCAGAAGACGGCATACGAGATTGCCTCTTGTCTCGTGGGCTCGGAGATGT
Ad2.12_GTAGAGGA	Ad2.12	rev	CAAGCAGAAGACGGCATACGAGATTCCTCTACGTCTCGTGGGCTCGGAGATGT
Ad2.13_GTCGTGAT	Ad2.13	rev	CAAGCAGAAGACGGCATACGAGATATCACGACGTCTCGTGGGCTCGGAGATGT
Ad2.14_ACCACTGT	Ad2.14	rev	CAAGCAGAAGACGGCATACGAGATACAGTGGTGTCTCGTGGGCTCGGAGATGT

Table S9. Sequencing files. Overview of NGS files and appearance in the figures.

ID number	Used for Figure	sample name	experiment	sequencing
ES_Input_ChIPseq	Fig 2: Input for peak finding	Input wt26	ChIP-seq	50bp PE
GS319	Fig 2C, 2D, 2E, 3B, 4A, 4D, 4F, 5B, 5F, 7A, 7B, S06, S09A, S11, S14, S16	FLAG-ChIP K14E8 Morc3-3xFLAG-KI	ChIP-seq	50bp PE
GS320	Fig 2C	FLAG-ChIP K14E8 Morc3-3xFLAG-KI	ChIP-seq	50bp PE
GS1024	Fig 2C	FLAG-ChIP K14E8 Morc3-3xFLAG-KI	ChIP-seq	50bp PE
GS1021	Fig 2C	FLAG-ChIP wt26	ChIP-seq	50bp PE
GS1023	Fig 2C	FLAG-ChIP wt26	ChIP-seq	50bp PE
GS180	Fig 2D, 2E	FLAG-ChIP K9-G1 Setdb1-3xFLAG-KI	ChIP-seq	50bp PE
Trim28 ChIP-seq	Fig 2D, 2E	GSM1819199, Published data set Yang et al. [9]	ChIP-seq	50bp PE
GS312	Fig 2D, 2E, 4A, 4B, 4F, S05F, S07A, S10C	H3K9me3-ChIP wt26	ChIP-seq	50bp PE
GS271	Fig 3A, 3F	RNAseq KO27-1	directional RNAseq	50bp PE
GS272	Fig 3A, 3F, 3B, 4F , S06, S16	RNAseq KO27-2	directional RNAseq	50bp PE
GS273	Fig 3A, 3F	RNAseq KO27-3	directional RNAseq	50bp PE
GS274	Fig 3A, 3F, S04A, S04B	RNAseq wt26 p42	directional RNAseq	50bp PE
GS275	Fig 3A, 3F, 3B, 4F , S04A, S04B, S06, S16	RNAseq wt26 p43	directional RNAseq	50bp PE
GS276	Fig 3A, 3F, S04A, S04B	RNAseq wt26 p56	directional RNAseq	50bp PE
GS948	Fig 3B, 4F, S04A, S04B, S06	RNAseq T131-3C	directional RNAseq	50 bpPE
GS947	Fig S04A, S04B	RNAseq wt26p46	directional RNAseq	50 bpPE
GS949	Fig S04A, S04B	RNAseq T131-3D	directional RNAseq	50 bpPE
GS950	Fig S04A, S04B	RNAseq T131-3M	directional RNAseq	50 bpPE
GS308	Fig 4A, 4B, 4D, 4F, S07A, S05F	H3K9me3-ChIP KO27-2	ChIP-seq	50bp PE
GS999	Fig 4A, 4B, S05F, S07A	H3K9me3-ChIP T131-3M	ChIP-seq	50bp PE
GS314	Fig 4B, S05F, S07A, S10C	H3K9me3-ChIP wt26	ChIP-seq	50bp PE
GS310	Fig 4B, S05F, S07A	H3K9m3-ChIP KO27-3	ChIP-seq	50bp PE
GS997	Fig 4B, 4F, S05F, S07A	H3K9me3-ChIP T131-3C	ChIP-seq	50bp PE
GS998	Fig 4B, S05F, S07A	H3K9me3-ChIP T131-3D	ChIP-seq	50bp PE
GS944	Fig 4C, 4F, 5E, S05F, S07B, S10B, S10D, S11	ATAC wt26	Omni-ATAC-seq	50 bp SE
GS945	Fig 4C, 4F, S05F, S07B, S11	ATAC KO27-2	Omni-ATAC-seq	50 bp SE
GS946	Fig 4C, 4F, S05F, S07B, S11	ATAC T131-3C	Omni-ATAC-seq	50 bp SE
GS1026	Fig4C, 5E, S05F, S07B, S10B, S10D	ATAC wt26	Omni-ATAC-seq	50 bp SE
GS1027	Fig4C, S05F, S07B	ATAC KO27-2	Omni-ATAC-seq	50 bp SE
GS1028	Fig4C, S05F, S07B	ATAC T131-3C	Omni-ATAC-seq	50 bp SE
GS1033	Fig4C, 5E, S05F, S07B, S10B, S10D	ATAC wt26	Omni-ATAC-seq	50 bp SE
GS1034	Fig4C, S05F, S07B	ATAC KO27-2	Omni-ATAC-seq	50 bp SE
GS1035	Fig4C, S05F, S07B	ATAC T131-3C	Omni-ATAC-seq	50 bp SE
GS1326	Fig 4D, 4F, S05B, S05F, S06, S10C	H3K27ac-ChIP wt26	ChIP-seq	50 bp SE
GS1327	Fig 4D, 4F, S05B, S05F, S06	H3K27ac-ChIP KO27-2	ChIP-seq	50 bp SE
GS1328	Fig 4D, 4F, S05B, S05F, S06	H3K27ac-ChIP T131-3C	ChIP-seq	50 bp SE
GS1065	Fig S08E	FLAG-ChIP T145-5 Morc3-WT-3xFLAG rescue	ChIPseq	50 bp PE
GS1066	Fig S08E	FLAG-ChIP T146-5 Morc3 ATP hydro E35A mut-3xFLAG rescue	ChIPseq	50 bp PE
GS1067	Fig S08E	FLAG-ChIP T148-3 Morc3 ATP bind G101A mut-3xFLAG rescue	ChIPseq	50 bp PE
GS1068	Fig S08E	FLAG-ChIP T149-3 Morc3 CW W419A mut-3xFLAG rescue	ChIPseq	50 bp PE
GS1069	Fig 5B, S08E, S09A	FLAG-ChIP T145-5 Morc3 WT-3xFLAG rescue	ChIPseq	50 bp PE
GS1070	Fig 5B, S08E, S09A	FLAG-ChIP T146-5 Morc3 ATP hydro E35A mut-3xFLAG rescue	ChIPseq	50 bp PE
GS1071	Fig 5B, S08E, S09A	FLAG-ChIP T148-3 Morc3 ATP bind G101A mut-3xFLAG rescue	ChIPseq	50 bp PE
GS1072	Fig 5B, S08E, S09A	FLAG-ChIP T149-3 Morc3 CW W419A mut-3xFLAG rescue	ChIPseq	50 bp PE
GS1322	Fig 5B, S08E, S09A	FLAG-ChIP T181-7J Morc3 SUMO mut-3xFLAG rescue	ChIPseq	50bp PE
GS1323	Fig 5B, S08E, S09A	FLAG-ChIP T185-5 Morc3 delta N mut-3xFLAG rescue	ChIPseq	50bp PE
GS311	Fig S09A, S09B	H3K4me3-ChIP wt26	ChIPseq	50bp PE
GS307	Fig S09A, S09B	H3K4me3 ChIP KO27-2	ChIPseq	50bp PE
GS1029	Fig 5E, S10B, S10D	ATAC T141-5	Omni-ATAC-seq	50 bp SE
GS1030	Fig 5E, S10B, S10D	ATAC T142-5	Omni-ATAC-seq	50 bp SE
GS1031	Fig 5E, S10B, S10D	ATAC T143-5	Omni-ATAC-seq	50 bp SE
GS1036	Fig 5E, S10B, S10D, S11	ATAC T141-5	Omni-ATAC-seq	50 bp SE
GS1037	Fig 5E, S10B, S10D, S11	ATAC T142-5	Omni-ATAC-seq	50 bp SE
GS1038	Fig 5E, S10B, S10D, S11	ATAC T143-5	Omni-ATAC-seq	50 bp SE
GS1324	Fig 5E, S10B, S10D, S11	ATAC T181-7J	Omni-ATAC-seq	50 bp SE
GS1325	Fig 5E, S10B, S10D, S11	ATAC T185-5	Omni-ATAC-seq	50 bp SE
GS1329	Fig 5E, S10B, S10D	ATAC T181-7J	Omni-ATAC-seq	50 bp SE
GS1330	Fig 5E, S10B, S10D	ATAC T185-5	Omni-ATAC-seq	50 bp SE
GS1337	Fig 4B, 5F, S10C	H3K9me3-ChIP wt26	ChIPseq	50bp PE
GS1338	Fig 4B, 5F, S10C	H3K9me3-ChIP KO27-2	ChIPseq	50bp PE
GS1339	Fig 4B, 5F, S10C	H3K9me3-ChIP T131-3C	ChIPseq	50bp PE
GS1340	Fig 5F, S10C	H3K9me3-ChIP T141-5	ChIPseq	50bp PE
GS1341	Fig 5F, S10C	H3K9me3-ChIP T142-5	ChIPseq	50bp PE
GS1342	Fig 5F, S10C	H3K9me3-ChIP T143-5	ChIPseq	50bp PE
GS1343	Fig 5F, S10C	H3K9me3-ChIP T181-7J	ChIPseq	50bp PE
GS1344	Fig 5F, S10C	H3K9me3-ChIP T185-5	ChIPseq	50bp PE
GS1243	Fig 7A, 7C, 7D, 7E, S13A, S13B	H3.3-ChIP wt26	ChIPseq	50bp PE
GS1244	Fig 7A, 7C, 7D, 7E, S13A, S13B	H3.3-ChIP KO27-2	ChIPseq	50bp PE
GS1306	Fig 7A, 7B, 7C, 7D, 7E, S13A, S13B, S14	H3.3-ChIP wt26	ChIPseq	50bp PE
GS1309	Fig 7A, 7B, 7C, 7D, 7E, S13A, S13B, S14	H3.3-ChIP KO27-2	ChIPseq	50bp PE
GS1310	Fig 7A, 7B, 7C, 7D, 7E, S13A, S13B, S14	H3.3-ChIP T131-3C	ChIPseq	50bp PE
GS1311	Fig 7A, 7B, S13A, S13B, S14	H3.3-ChIP T141-5	ChIPseq	50bp PE
GS1312	Fig 7A, 7B, S13A, S13B, S14	H3.3-ChIP T142-5	ChIPseq	50bp PE
GS1313	Fig 7A, 7B, S13A, S13B, S14	H3.3-ChIP T143-5	ChIPseq	50bp PE
GS1314	Fig 7A, 7B, S13A, S13B, S14	H3.3-ChIP T181-7J	ChIPseq	50bp PE
GS1315	Fig 7A, 7B, S13A, S13B, S14	H3.3-ChIP T185-5	ChIPseq	50bp PE
Daxx.res.1 RNAseq	S15, S16	GSM2742946, published data set Hoelper et al. [2]	RNAseq	100bp SE
Daxx.res.2 RNAseq	S15	GSM2742947, published data set Hoelper et al. [2]	RNAseq	100bp SE
Daxx.ko.1 RNAseq	S15, S16	GSM2742944, published data set Hoelper et al. [2]	RNAseq	100bp SE
Daxx.ko.2 RNAseq	S15	GSM2742945, published data set Hoelper et al. [2]	RNAseq	100bp SE
H3.3.res.1 RNAseq	S15, S16	GSM2742950, published data set Hoelper et al. [2]	RNAseq	100bp SE
H3.3.res.2 RNAseq	S15	GSM2742951, published data set Hoelper et al. [2]	RNAseq	100bp SE
H3.3.ko.1 RNAseq	S15, S16	GSM2742948, published data set Hoelper et al. [2]	RNAseq	100bp SE
H3.3.ko.2 RNAseq	S15	GSM2742949, published data set Hoelper et al. [2]	RNAseq	100bp SE

Table S10. Software

Name	Reference
Bowtie2 v2.2.9	Langmead & Salzberg [10]
Homer v4.9	Heinz et al. [11]
Samtools v1.9	Li et al. [12]
Bedtools v2.28.0	Quinlan & Hall [13]
RepEnrich2 v2.7	Criscione et al. [14]
DeSeq2 v1.30.0	Love et al. [15]
IGV 2.10.0	Robinson et al.[16]
STAR v2.7	Dobin et al. [17]
Picard v2.17.11	Broad Institute [18]
Trimmomatic 0.36	Bolger [19]
RigerJ v2.02	Broad Institute [20]
Panther v16.0	Thomas et al. [21]
ImageJ	Schneider et al. [22]
Maxquant v1.6.14.0	Cox & Mann [23]

References

1. Chelmicki, T. et al. m(6)A RNA methylation regulates the fate of endogenous retroviruses. Nature 591, 312-316, doi:10.1038/s41586-020-03135-1 (2021).
2. Hoelper, D., Huang, H., Jain, A. Y., Patel, D. J. & Lewis, P. W. Structural and mechanistic insights into ATRX-dependent and -independent functions of the histone chaperone DAXX. Nat Commun 8, 1193, doi:10.1038/s41467-017-01206-y (2017)
3. Peters, A.H. et al. Loss of the Suv39h histone methyltransferases impairs mammalian heterochromatin and genome stability. Cell. 107(3):323-37. doi: 10.1016/s0092-8674(01)00542-6 (2001)
4. Nakashima, T. et al. Evidence for osteocyte regulation of bone homeostasis through RANKL expression. Nat Med.17(10):1231-4. doi: 10.1038/nm.2452. (2011)
5. Sadic D. et al. Atrx promotes heterochromatin formation at retrotransposons. EMBO Rep.16(7):836-50. doi: 10.15252/embr.201439937. (2015)
6. Rowe, H.M. et al. KAP1 controls endogenous retroviruses in embryonic stem cells. Nature 463, 237-240. 10.1038/nature08674. (2010)
7. Maksakova, I.A. et al. Distinct roles of KAP1, HP1 and G9a/GLP in silencing of the two-cell-specific retrotransposon MERVL in mouse ES cells. Epigenetics Chromatin. 6(1):15. doi: 10.1186/1756-8935-6-15. (2013)
8. Buenrostro, J.D., Giresi, P.G., Zaba, L.C., Chang, H.Y., and Greenleaf, W.J. Transposition of native chromatin for fast and sensitive epigenomic profiling of open chromatin, DNA-binding proteins and nucleosome position. Nat Methods 10, 1213-1218. 10.1038/nmeth.2688. (2013)
9. Yang, B.X., et al. Systematic identification of factors for provirus silencing in embryonic stem cells. Cell. 163(1):230-45. doi: 10.1016/j.cell.2015.08.037. (2015)
10. Langmead, B., Salzberg, S. Fast gapped-read alignment with Bowtie 2. Nat Methods 9, 357–359 (2012)
11. Heinz, S. et al. Simple Combinations of Lineage-Determining Transcription Factors Prime cis-Regulatory Elements Required for Macrophage and B Cell Identities. Mol Cell ;38(4):576-589 (2010)
12. Li H., et al. The Sequence alignment/map (SAM) format and SAMtools. Bioinformatics, 25, 2078-9. (2009)
13. Quinlan, A.R. and Hall, I.M. BEDTools: a flexible suite of utilities for comparing genomic features, Bioinformatics, 26, 841–842, https://doi.org/10.1093/bioinformatics/btq033 (2010)
14. Criscione, S.W. et al. Transcriptional landscape of repetitive elements in normal and cancer human cells. BMC Genomics 15, 583 .https://doi.org/10.1186/1471-2164-15-583 (2014)
15. Love, M.I., Huber, W., Anders, S. Moderated estimation of fold change and dispersion forRNA-seq data with DESeq2. Genome Biology, 15:550.https://doi.org/10.1186/s13059-014-0550-8 (2014)
16. Robinson, J.T. Integrative Genomics Viewer. Nature Biotechnology 29, 24–26 (2011).
17. Dobin, A. STAR: ultrafast universal RNA-seq aligner, Bioinformatics, 29, (2013)
18. “Picard Toolkit.”. Broad Institute, GitHub Repository. http://broadinstitute.github.io/picard/; Broad Institute (2019)
19. Bolger, A. M., Lohse, M., & Usadel, B. Trimmomatic: A flexible trimmer for Illumina Sequence Data. Bioinformatics, btu170. (2014)
20. “rigerj” Broad Institute, GitHub Repository. https://github.com/broadinstitute/rigerj ; Broad Institute (2017)
21. Thomas, P.D. PANTHER: a library of protein families and subfamilies indexed by function. Genome Res., 13: 2129-2141 .(2003)
22. Schneider, C. A.; Rasband, W. S. & Eliceiri, K. W. "NIH Image to ImageJ: 25 years of image analysis", Nature methods 9(7): 671-675, (2012)
23. Cox, J. and Mann, M. MaxQuant enables high peptide identification rates, individualized p.p.b.-range mass accuracies and proteome-wide protein quantification. Nat Biotechnol, 26, 1367-72. (2008)
