# Supplementary material for: Morc3 silences endogenous retroviruses by enabling Daxx-mediated histone H3.3 incorporation
Source: Nat Commun. 2021 Oct 14;12:5996. doi: 10.1038/s41467-021-26288-7 (PMC8516933; doi:10.1038/s41467-021-26288-7)
Supplement: Supplementary file 3 — Description of Additional Supplementary Files [file 41467_2021_26288_MOESM3_ESM.pdf]

Description of Additional Supplementary Files

File Name: Supplementary Data 1
Description: Results of sgRNA screen.

File Name: Supplementary Data 2
Description: Morc3 ChIP-seq peaks.

File Name: Supplementary Data 3
Description: Morc3 ChIP-seq enrichment over input on ERVs.

File Name: Supplementary Data 4
Description: Gene expression changes in Morc3 ko cells.

File Name: Supplementary Data 5
Description: Morc3 target genes.

File Name: Supplementary Data 6
Description: ERV expression changes in Morc3 ko cells.

File Name: Supplementary Data 7
Description: Morc3 ChIP-MS results.
