# Supplementary material for: Morc3 silences endogenous retroviruses by enabling Daxx-mediated histone H3.3 incorporation
Source: Nat Commun. 2021 Oct 14;12:5996. doi: 10.1038/s41467-021-26288-7 (PMC8516933; doi:10.1038/s41467-021-26288-7)
Supplement: Supplementary file 11 — Reporting Summary [file 41467_2021_26288_MOESM11_ESM.pdf]

nature research

Corresponding author(s): Gunnar Schotta

Last updated by author(s): Sep 8, 2021

Reporting Summary

Nature Research wishes to improve the reproducibility of the work that we publish. This form provides structure for consistency and transparency in reporting. For further information on Nature Research policies, see our Editorial Policies and the Editorial Policy Checklist.

Statistics

For all statistical analyses, confirm that the following items are present in the figure legend, table legend, main text, or Methods section.

n/a	Confirmed	
☐	☒	The exact sample size (*n*) for each experimental group/condition, given as a discrete number and unit of measurement
☐	☒	A statement on whether measurements were taken from distinct samples or whether the same sample was measured repeatedly
☐	☒	The statistical test(s) used AND whether they are one- or two-sided<br>*Only common tests should be described solely by name; describe more complex techniques in the Methods section.*
☒	☐	A description of all covariates tested
☒	☐	A description of any assumptions or corrections, such as tests of normality and adjustment for multiple comparisons
☐	☒	A full description of the statistical parameters including central tendency (e.g. means) or other basic estimates (e.g. regression coefficient) AND variation (e.g. standard deviation) or associated estimates of uncertainty (e.g. confidence intervals)
☐	☒	For null hypothesis testing, the test statistic (e.g. *F*, *t*, *r*) with confidence intervals, effect sizes, degrees of freedom and *P* value noted<br>*Give P values as exact values whenever suitable.*
☒	☐	For Bayesian analysis, information on the choice of priors and Markov chain Monte Carlo settings
☒	☐	For hierarchical and complex designs, identification of the appropriate level for tests and full reporting of outcomes
☒	☐	Estimates of effect sizes (e.g. Cohen's *d*, Pearson's *r*), indicating how they were calculated

Our web collection on statistics for biologists contains articles on many of the points above.

Software and code

Policy information about availability of computer code

Data collection	Flow cytometry: BD FACS Diva v8.0.1, RT-qPCR: Roche LightCycler480 v1.5.1
Data analysis	FlowJo v7.6.1, bowtie1 v1.1.2, R v3.5.1 & v3.6.0, bedtools2 v2.28.0, bowtie2 v2.2.9, homer v4.9, samtools v1.9, RepEnrich2 v2.7, DeSeq2 v1.30.0, IGV v2.10, STAR v2.7.1a, Picard tools v 2.17.11, Trimmomatic v0.36, RigerJ v2.0.2, Panther v16.0, ImageJ, Maxquant v1.6.14.0, LIMMA, licor image studio lite v5.2

For manuscripts utilizing custom algorithms or software that are central to the research but not yet described in published literature, software must be made available to editors and reviewers. We strongly encourage code deposition in a community repository (e.g. GitHub). See the Nature Research guidelines for submitting code & software for further information.

Data

Policy information about availability of data

All manuscripts must include a data availability statement. This statement should provide the following information, where applicable:

- Accession codes, unique identifiers, or web links for publicly available datasets
- A list of figures that have associated raw data
- A description of any restrictions on data availability

ATAC-seq, ChIP-seq and RNA-seq datasets were deposited to GEO (accession GSE159936).
ChIP-MS proteomics datasets were deposited to ProteomeXchange (accession PXD027368).

Field-specific reporting

Please select the one below that is the best fit for your research. If you are not sure, read the appropriate sections before making your selection.

☒ Life sciences ☐ Behavioural & social sciences ☐ Ecological, evolutionary & environmental sciences

For a reference copy of the document with all sections, see nature.com/documents/nr-reporting-summary-flat.pdf

Life sciences study design

All studies must disclose on these points even when the disclosure is negative.

Sample size	Sequencing experiments were perfomed in three or two biological replicates. Details are listed in table S9.
Data exclusions	No data exclusions
Replication	Replications were successful, 2 or more replicates were performed.
Randomization	No randomization. Not relevant to this study since the data was processed through a standardized analysis that excluded human bias.
Blinding	No blinding. Not relevant to this study since the data was processed through a standardized analysis that excluded human bias.

Reporting for specific materials, systems and methods

We require information from authors about some types of materials, experimental systems and methods used in many studies. Here, indicate whether each material, system or method listed is relevant to your study. If you are not sure if a list item applies to your research, read the appropriate section before selecting a response.

Materials & experimental systems

n/a	Involved in the study	
☐	☒	Antibodies
☐	☒	Eukaryotic cell lines
☒	☐	Palaeontology and archaeology
☒	☐	Animals and other organisms
☒	☐	Human research participants
☒	☐	Clinical data
☒	☐	Dual use research of concern

Methods

n/a	Involved in the study	
☐	☒	ChIP-seq
☐	☒	Flow cytometry
☒	☐	MRI-based neuroimaging

Antibodies

Antibodies used	NP95 (clone 8H3, gift from H. Leonhardt), Nanog (Biomol, A300-397A) FLAG M2 (Sigma, F1804), H3K9me3 (Active Motif, 39161), LaminB (Active motif, 39095), H3K4me3 (Diagenode, CS-003-100), H3K27ac (Diagenode, pAB-174-050), Morc3 (Rockland, 100-401-N96S), Fibrillarin (Santa Cruz, sc-166021), Daxx (Santa Cruz, sc-8043), Histone H3.3 (Millipore, 09-838)
Validation	Validation statements on the manufacturer's websites: Nanog https://www.biomol.com/de/produkte/antikoerper/primaerantikoerper/allgemein/anti-nanog-a300-397a-tFLAG M2 https://www.sigmaaldrich.com/DE/de/product/sigma/f1804H3K9me3 https://www.activemotif.com/catalog/details/39161LaminB1 https://www.activemotif.com/catalog/details/39095/lamin-b1-antibody-pabH3K4me3 https://www.diagenode.com/en/p/h3k4me3-polyclonal-antibody-classic-100-ulH3K27ac https://www.diagenode.com/en/p/h3k27ac-polyclonal-antibody-classic-50-mg-42-mlMorc3 https://rockland-inc.com/Product.aspx?id=44712Fibrillarin https://www.scbt.com/p/fibrillarin-antibody-g-4Daxx (H-7) https://www.scbt.com/p/daxx-antibody-h-7Histone H3.3 https://www.merckmillipore.com/DE/de/product/Anti-Histone-H3.3-Antibody,MM_NF-09-838

Eukaryotic cell lines

Policy information about cell lines

Cell line source(s)	wt26:Peters et al, 2001(DOI:https://doi.org/10.1016/S0092-8674(01)00542-6), A9:Nakashima et al., 2011 (DOI: https://doi.org/10.1038/nm.2452), T86:Sadic et al., 2015(DOI: https://doi.org/10.15252/embr.201439937) , T37:Sadic et al., 2015, 293T:Invitrogen, T90:This study, T89:This study, KO27-1:This study, KO27-2:This study, KO27-3:This study, K9-G1:Schotta lab, K14-E8:This study, T90-B1:This study, T90-B2:This study, T90-D1:This study, KO43-4:This study, KO43-7:This study, KO43-8:This study, T131-3C:This study, T131-3D:This study, T131-3M:This study, T141-5:This study, T141-7:This study, T141-9:This study, T142-4:This study, T142-5:This study, T142-11:This study, T143-2:This study, T143-3:This study, T143-5:This study, T145-5:This study, T146-5:This study, T148-3:This study, T149-3:This study, T181-6D:This study, T181-7J:This study, T181-15:This study, T185-5:This study, T185-7:This study, T185-12:This study, KO2-3:Sadic et al., 2015, T163-8:This study, T163-4:This study, T163-9:This study, T164-3:This study, T164-7:This study, T164-8:This study
Authentication	PCR, Westernblot, FACS staining
Mycoplasma contamination	All cell lines are tested negative for mycoplasma contamination
Commonly misidentified lines (See ICLAC register)	None

ChIP-seq

Data deposition

- [x] Confirm that both raw and final processed data have been deposited in a public database such as GEO.
- [x] Confirm that you have deposited or provided access to graph files (e.g. BED files) for the called peaks.

Data access links *May remain private before publication.*	GSE159936
Files in database submission	ES_Input_ChIPseq, GS319, GS320, GS1024, GS1021, GS1023, GS180, GS312, GS271, GS272, GS273, GS274, GS275, GS276, GS948, GS947, GS949, GS950, GS308, GS999, GS314, GS310, GS997, GS998, GS944, GS945, GS946, GS1026, GS1027, GS1028, GS1033, GS1034, GS1035, GS1326, GS1327, GS1328, GS1065, GS1066, GS1067, GS1068, GS1069, GS1070, GS1071, GS1072, GS1322, GS1323, GS311, GS307, GS1029, GS1030, GS1031, GS1036, GS1037, GS1038, GS1324, GS1325, GS1329, GS1330, GS1337, GS1338, GS1339, GS1340, GS1341, GS1342, GS1343, GS1344, GS1243, GS1244, GS1306, GS1309, GS1310, GS1311, GS1312, GS1313, GS1314, GS1315
Genome browser session (e.g. UCSC)	No longer applicable

Methodology

Replicates	2-3 replicates
Sequencing depth	50bp PE, 20-30 Mio reads
Antibodies	FLAG M2 (Sigma, F1804), H3K9me3 (Active Motif, 39161), H3K4me3 (Diagenode, CS-003-100), H3K27ac (Diagenode, pAB-174-050), Histone H3.3 (Millipore,#09-838)
Peak calling parameters	bowtie2 -q -p 16 -x<br>Homer findPeaks -style factor
Data quality	fastqc
Software	Bowtie2, Homer, Samtools, Bedtools, RepEnrich2, DeSeq2

Flow Cytometry

Plots

Confirm that:

- [x] The axis labels state the marker and fluorochrome used (e.g. CD4-FITC).
- [x] The axis scales are clearly visible. Include numbers along axes only for bottom left plot of group (a 'group' is an analysis of identical markers).
- [x] All plots are contour plots with outliers or pseudocolor plots.
- [x] A numerical value for number of cells or percentage (with statistics) is provided.

Methodology

Sample preparation	mES cells in PBS
Instrument	BD FACS Canto
Software	BD FACSDiva, FlowJo
Cell population abundance	No sorting
Gating strategy	Gating for live cells on FSC/SSC, gating for negative and positive according to control cells without GFP expression

☒ Tick this box to confirm that a figure exemplifying the gating strategy is provided in the Supplementary Information.
