# Supplementary material for: Morc3 silences endogenous retroviruses by enabling Daxx-mediated histone H3.3 incorporation
Source: Nat Commun. 2021 Oct 14;12:5996. doi: 10.1038/s41467-021-26288-7 (PMC8516933; doi:10.1038/s41467-021-26288-7)
Supplement: Supplementary file 12 — Source Data [file 41467_2021_26288_MOESM12_ESM.zip › Source_data_folder/FigS02_Morc3_KI.pdf]

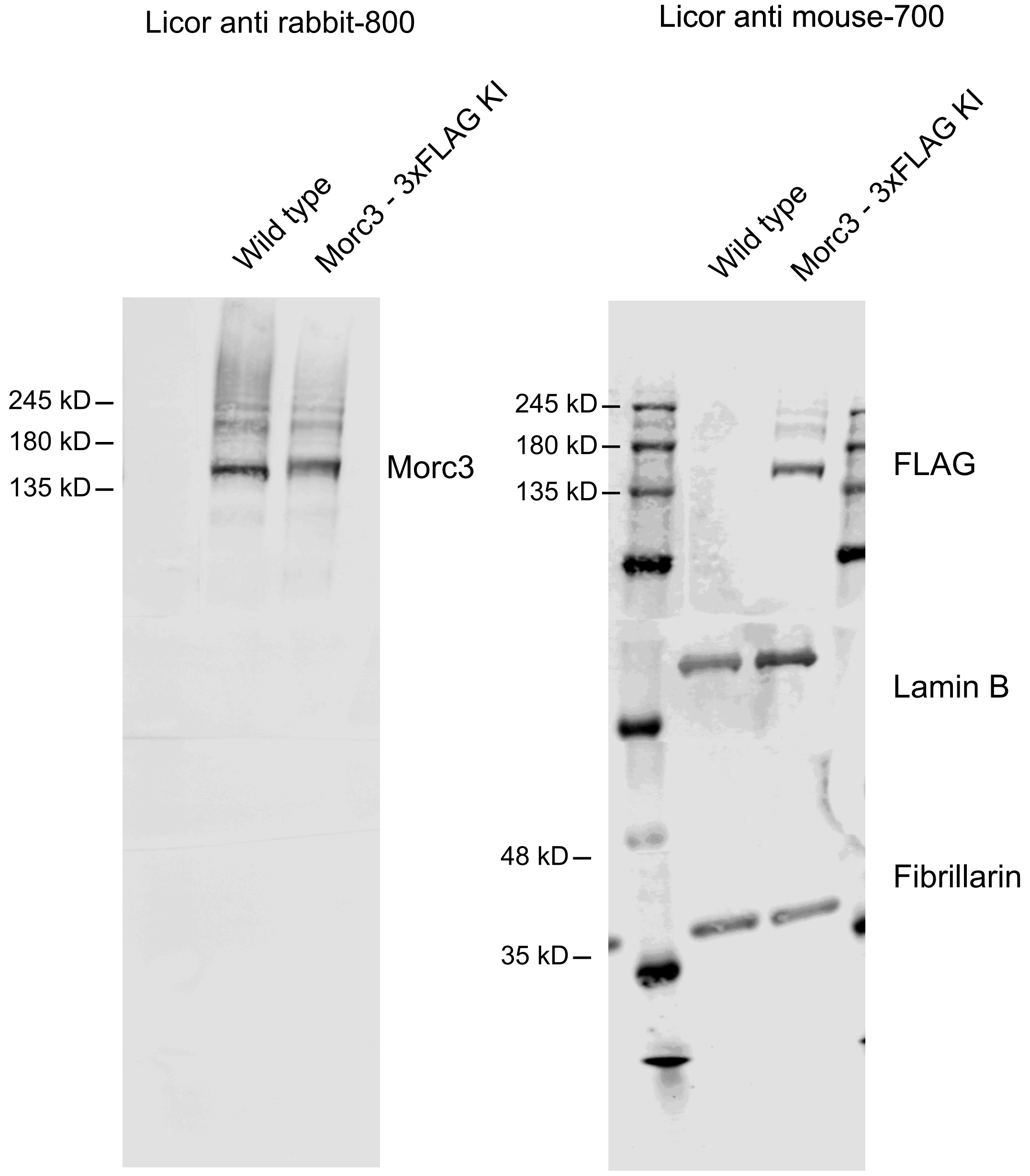

Licor anti rabbit-800
Licor anti mouse-700
Wild type
Morc3 - 3xFLAG KI
245 kD
180 kD
135 kD
Morc3
48 kD
35 kD
FLAG
Lamin B
Fibrillarin
