# Supplementary material for: Morc3 silences endogenous retroviruses by enabling Daxx-mediated histone H3.3 incorporation
Source: Nat Commun. 2021 Oct 14;12:5996. doi: 10.1038/s41467-021-26288-7 (PMC8516933; doi:10.1038/s41467-021-26288-7)
Supplement: Supplementary file 12 — Source Data [file 41467_2021_26288_MOESM12_ESM.zip › Source_data_folder/Fig6F_Daxx_IP_WB.pdf]

Licor anti mouse 700

No primary antibody stainig of upper part of the left membrane. Signal comes from mouse anti-FLAG antibody used in IP

Fibrillarin

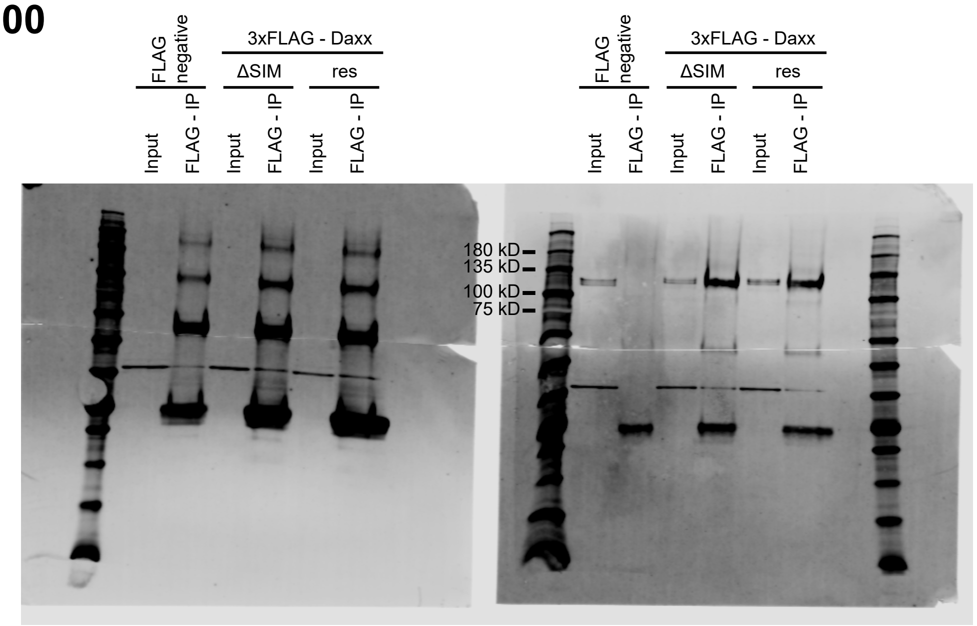

Daxx

Fibrillarin

Elution with beta-mercapto

Elution without beta-mercapto

Licor anti rabbit 800

Morc3

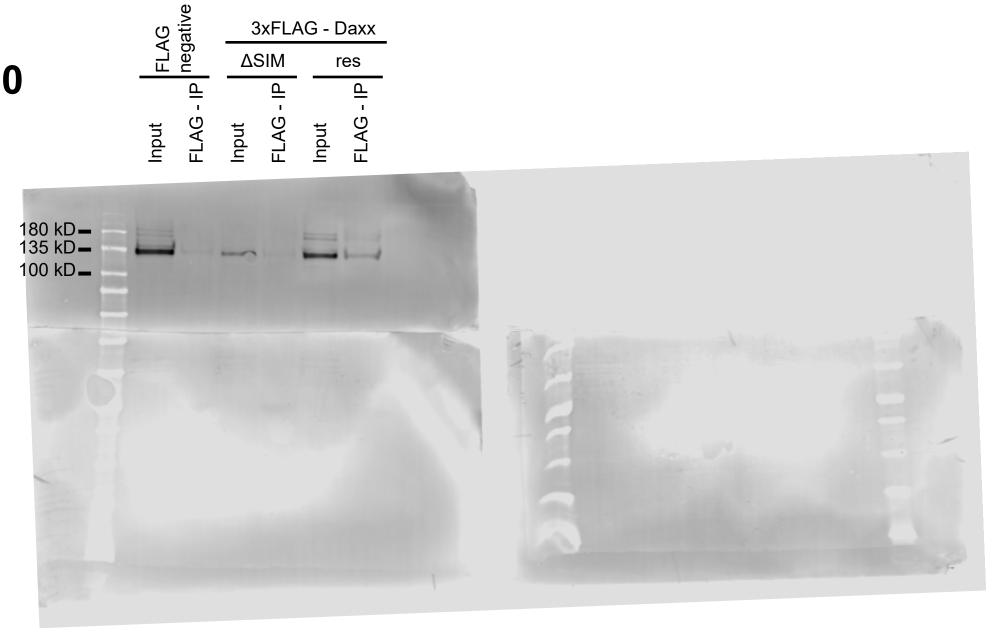

Elution with beta-mercapto

Elution without beta-mercapto

Reprobing of same membrane (only lower parts) with rabbit anti-Nanog antibody

Morc3

Nanog

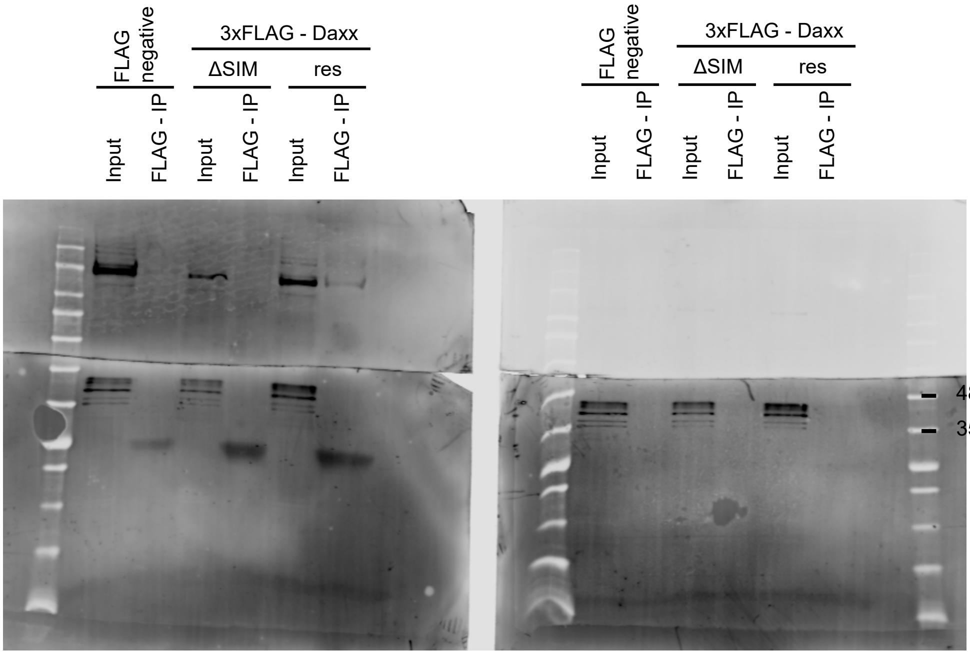

Nanog

Elution with beta-mercapto

Elution without beta-mercapto
