# Supplementary figures and images for: Morc3 silences endogenous retroviruses by enabling Daxx-mediated histone H3.3 incorporation
Source: Nat Commun. 2021 Oct 14;12:5996. doi: 10.1038/s41467-021-26288-7 (PMC8516933; doi:10.1038/s41467-021-26288-7)

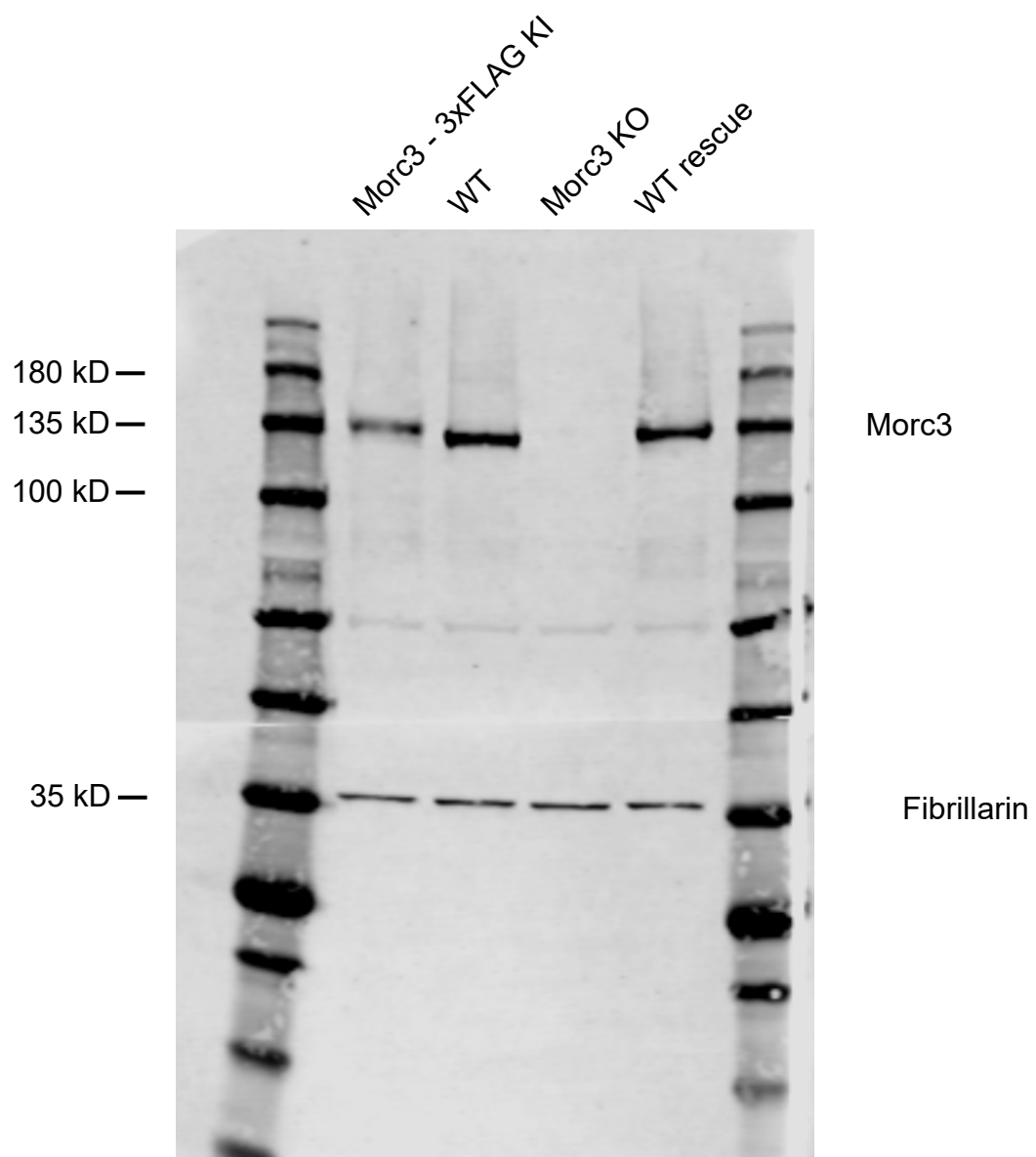

Supplement: Supplementary file 12 — Source Data [file 41467_2021_26288_MOESM12_ESM.zip › Source_data_folder/FigS03B_WB_Morc3_rescue.pdf]

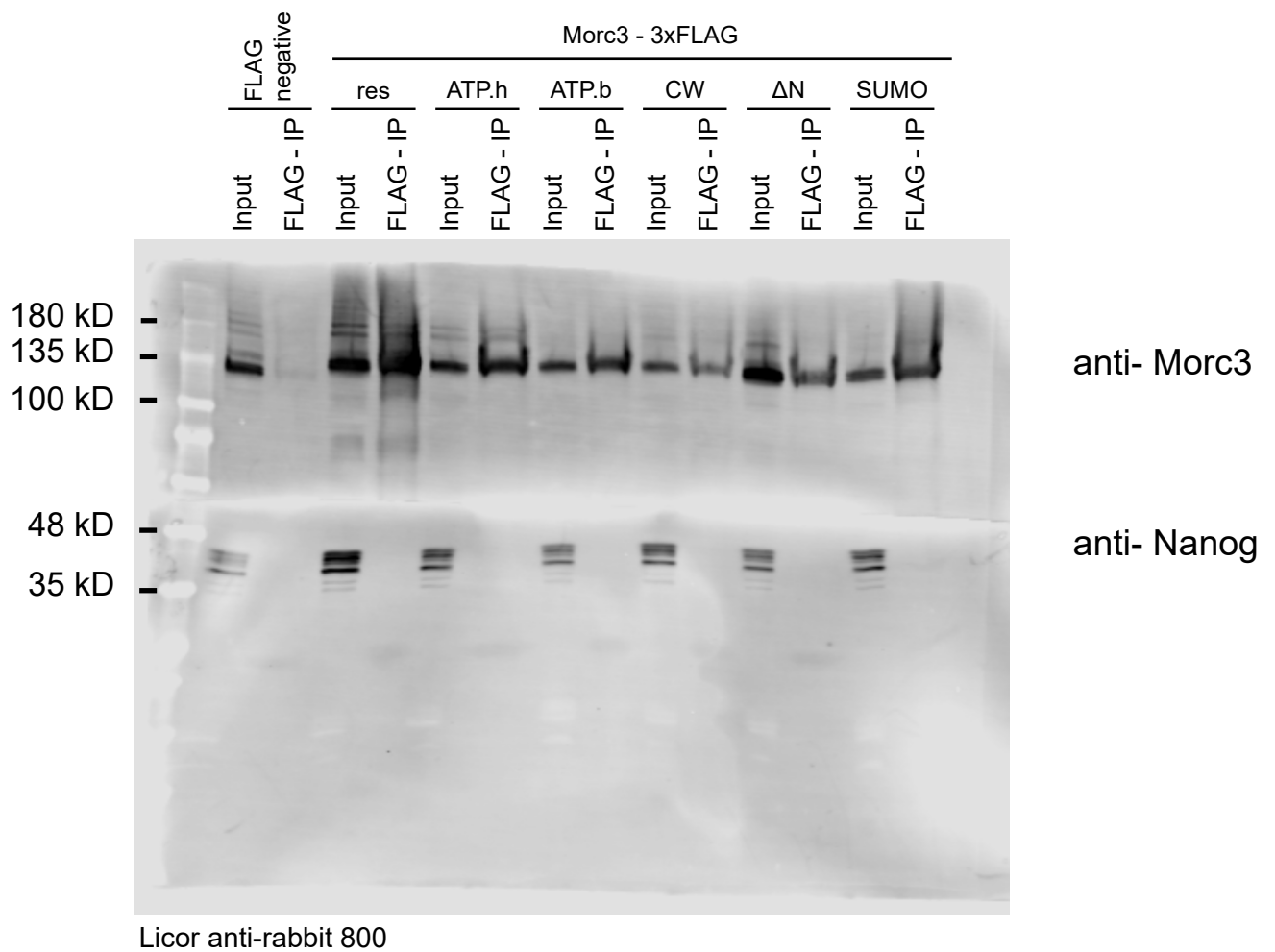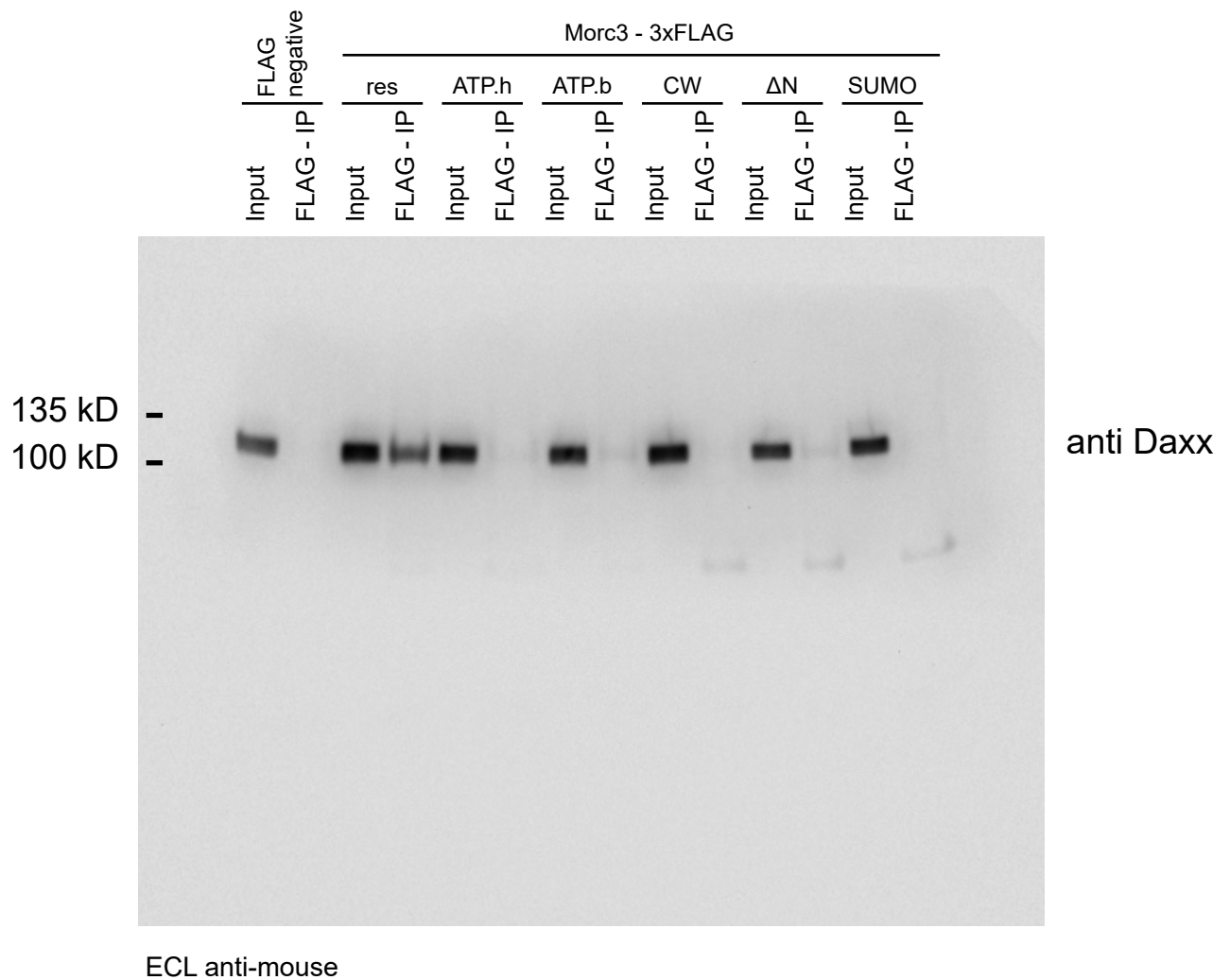

Supplement: Supplementary file 12 — Source Data [file 41467_2021_26288_MOESM12_ESM.zip › Source_data_folder/Fig6D_Morc3_IP_WB.pdf]

Licor anti-mouse 800

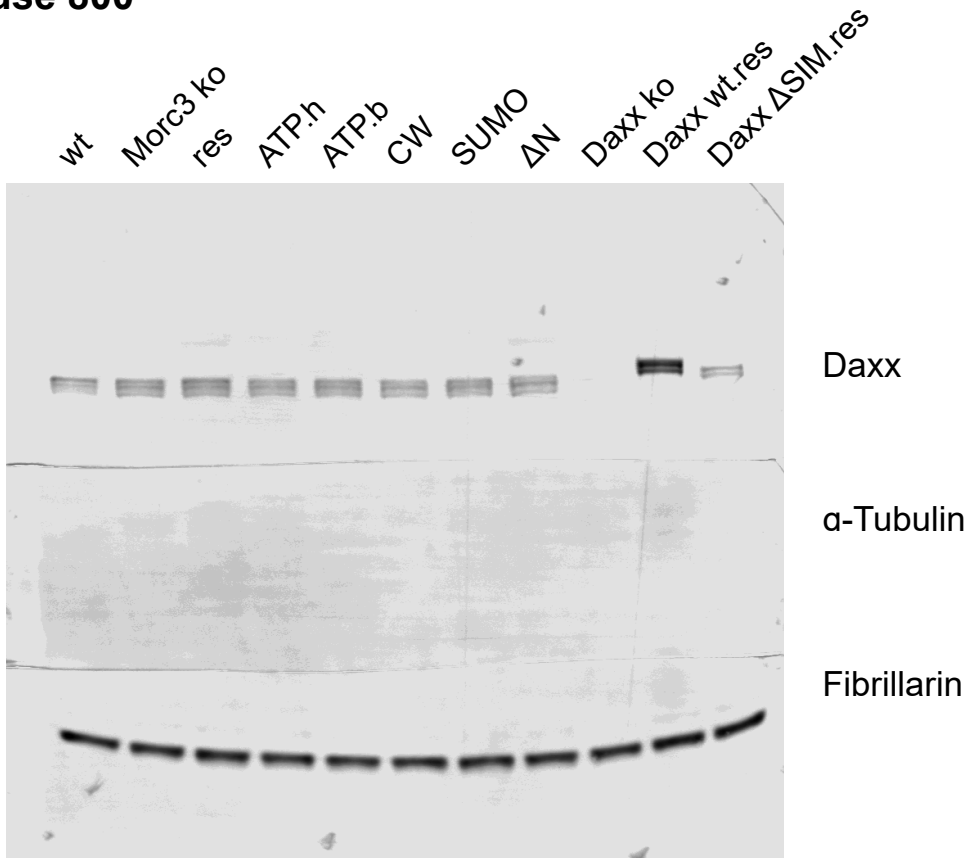

Licor anti-rabbit 700

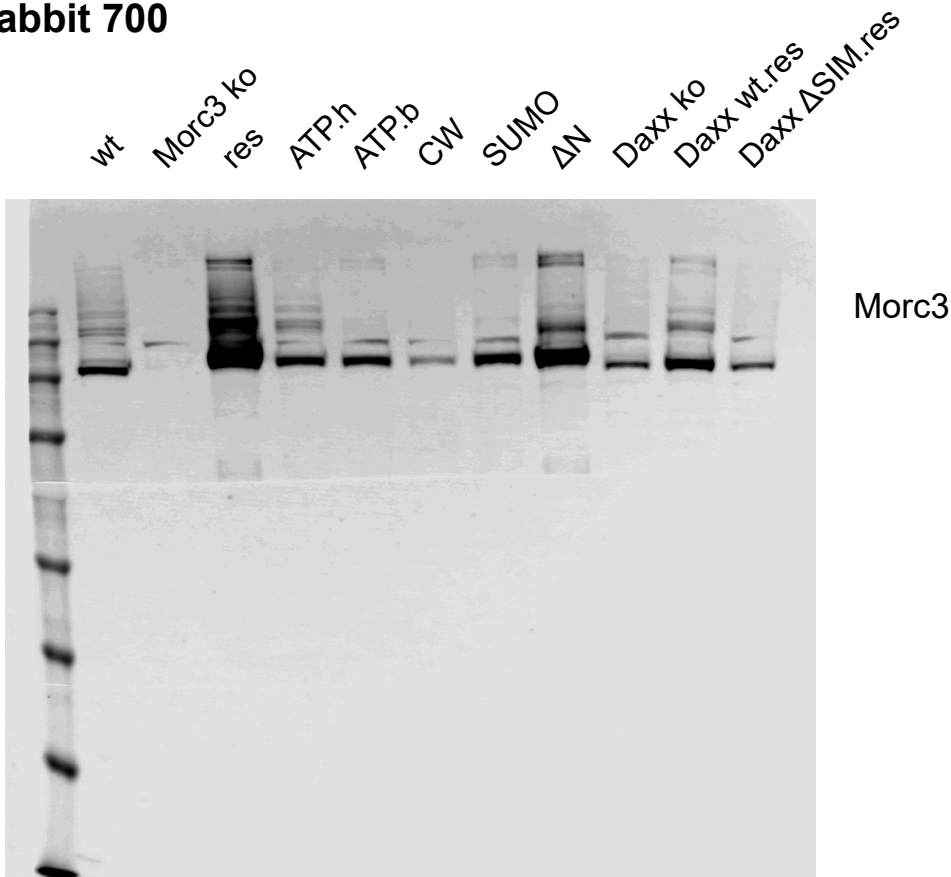

Supplement: Supplementary file 12 — Source Data [file 41467_2021_26288_MOESM12_ESM.zip › Source_data_folder/FigS12_WB_NE_Morc3_Daxx.pdf]

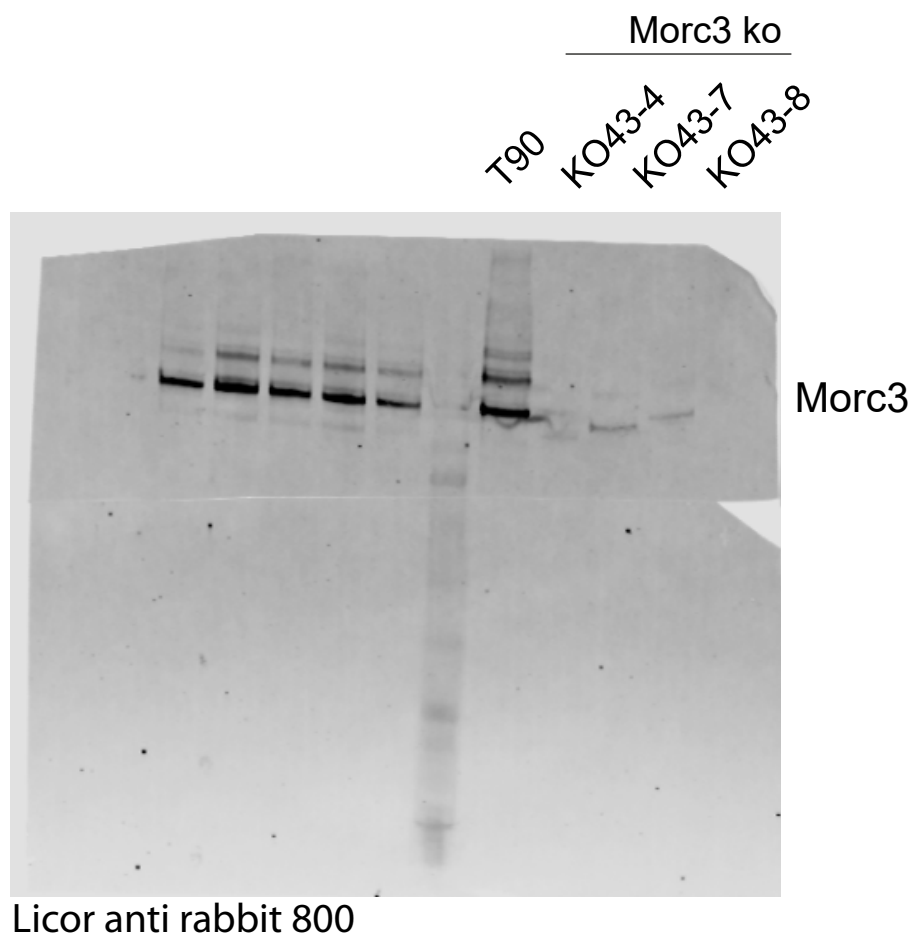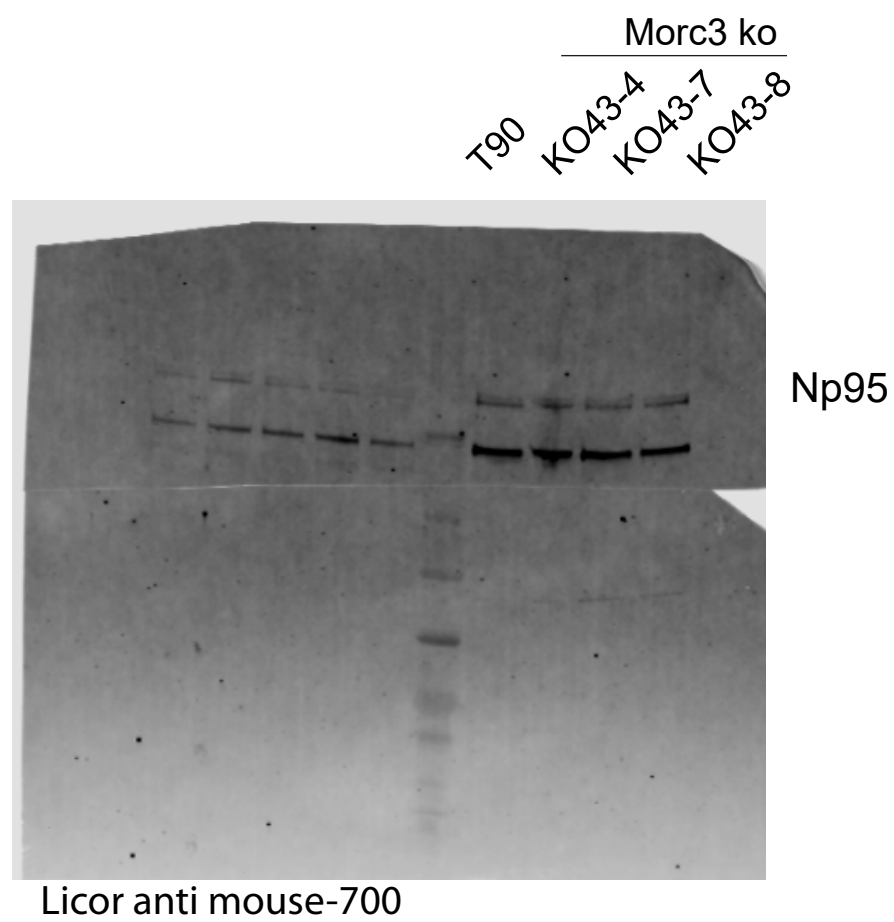

Supplement: Supplementary file 12 — Source Data [file 41467_2021_26288_MOESM12_ESM.zip › Source_data_folder/FigS03C_WB_Morc3_KO_T90.pdf]

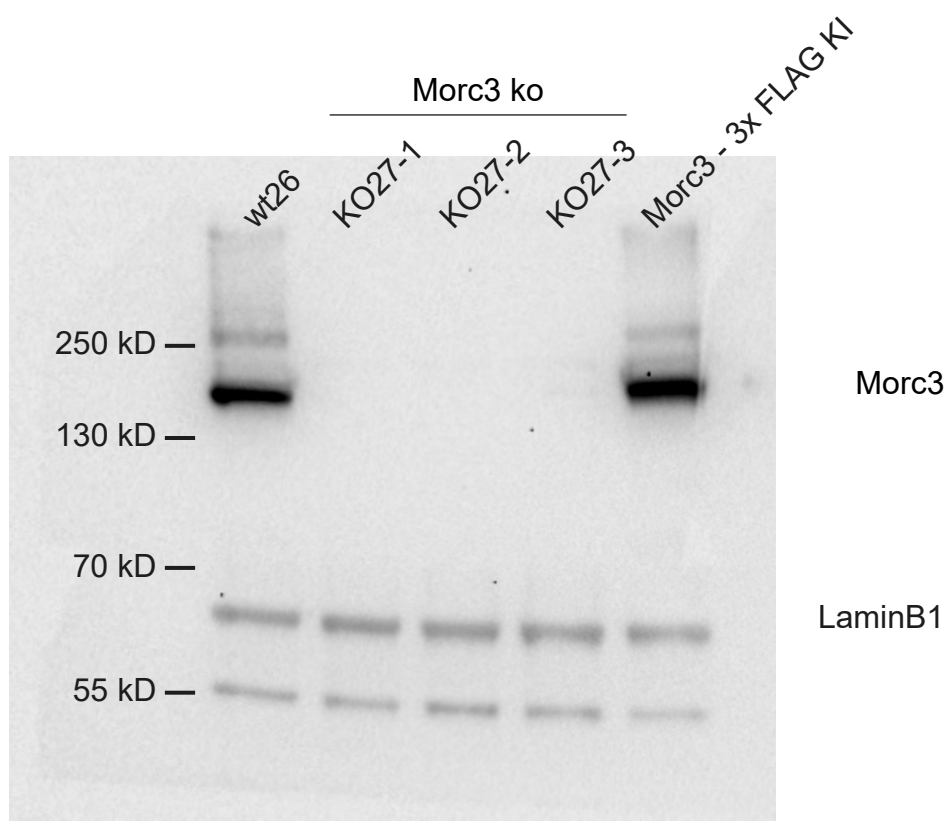

Supplement: Supplementary file 12 — Source Data [file 41467_2021_26288_MOESM12_ESM.zip › Source_data_folder/FigS03A_WB_KO.pdf]

Licor anti rabbit 800

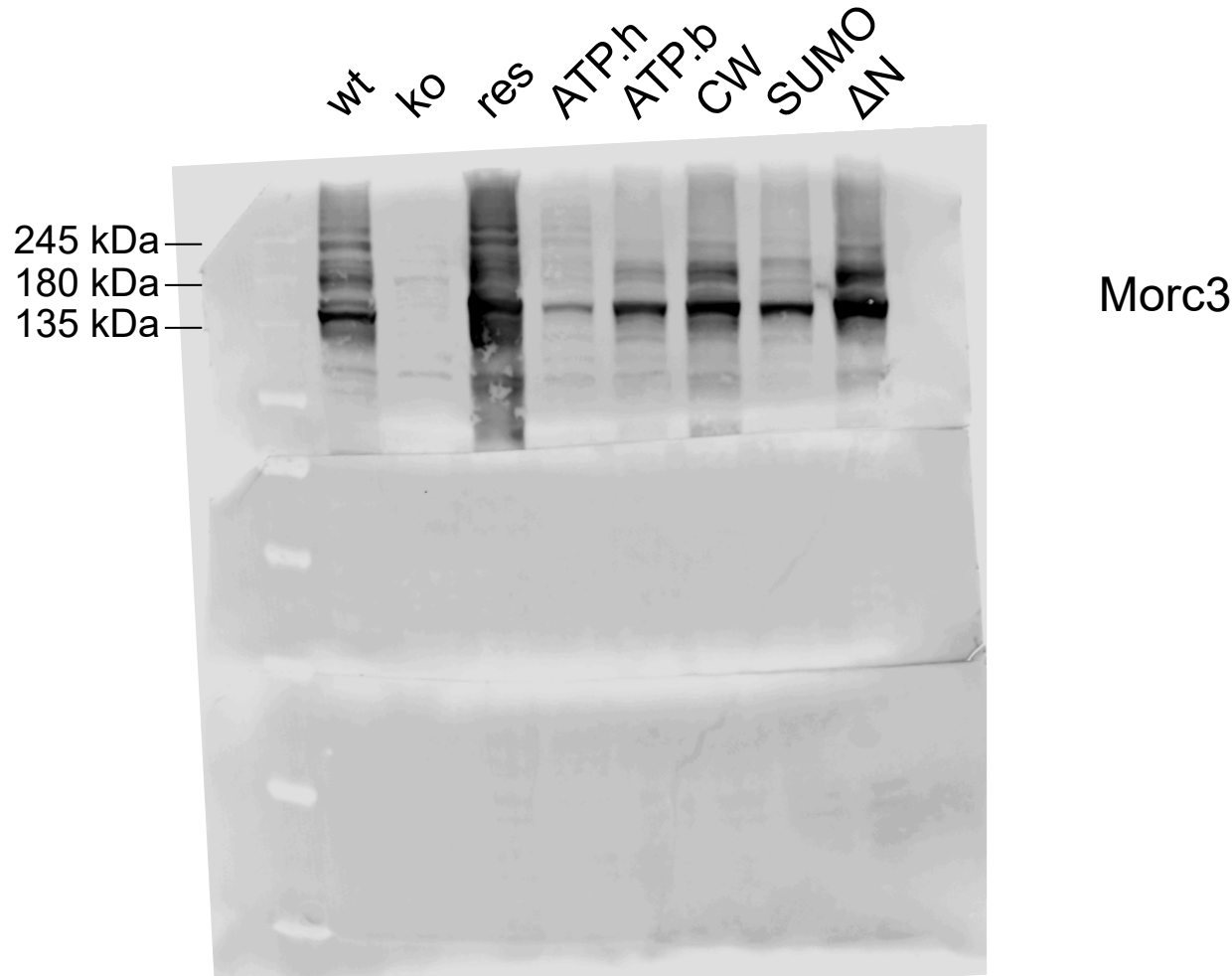

Licor anti mouse 700

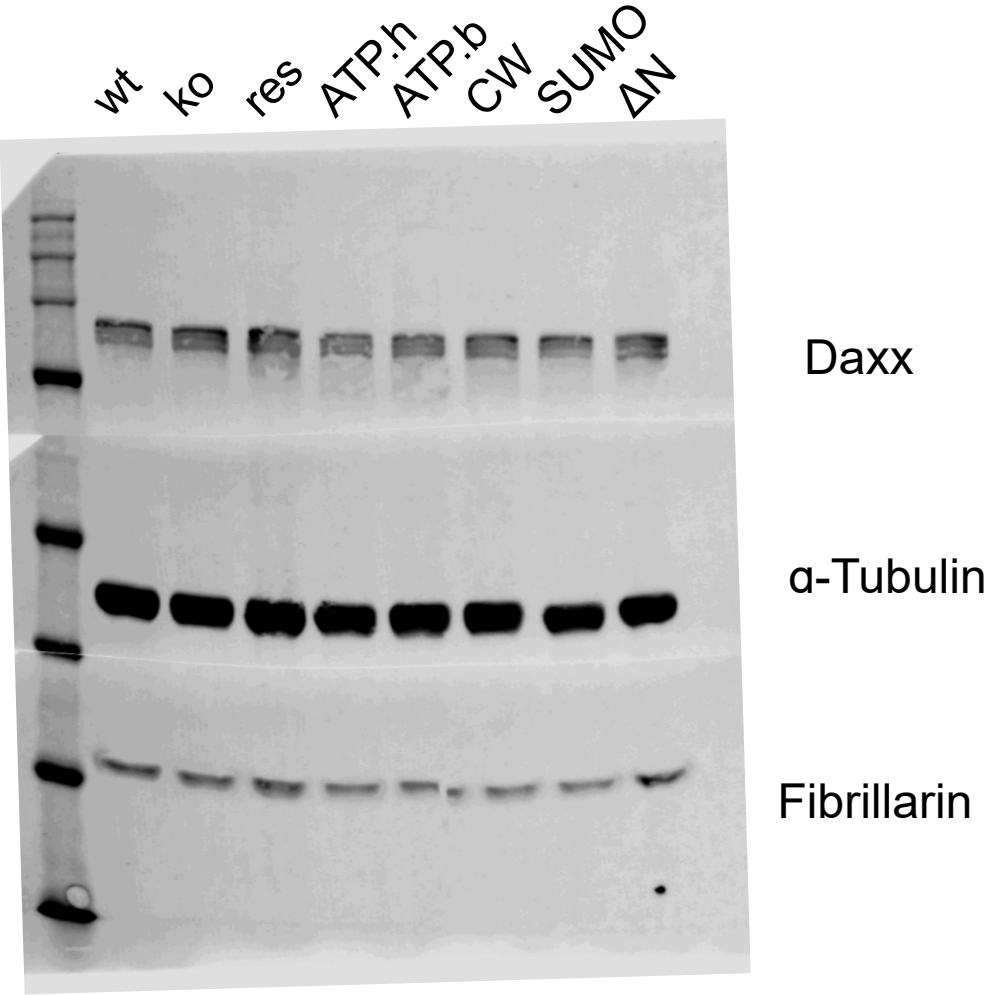

Supplement: Supplementary file 12 — Source Data [file 41467_2021_26288_MOESM12_ESM.zip › Source_data_folder/FigS08C_WB_WCL_Morc3_mutants.pdf]
